# Unveiling the Burden of Steatotic Liver Disease: Mortality Risks by Subtype and Fibrosis Stage in a Nationwide Cohort

**DOI:** 10.1111/liv.70485

**Published:** 2025-12-29

**Authors:** Qi Feng, Pinelopi Manousou, Chioma N. Izzi‐Engbeaya, Rohit Loomba, Mark Thursz, Mark Woodward

**Affiliations:** ^1^ The George Institute for Global Health (UK), School of Public Health, Faculty of Medicine Imperial College London London UK; ^2^ Division of Digestive Diseases, Department of Metabolism, Digestion and Reproduction, Faculty of Medicine Imperial College London London UK; ^3^ Department of Hepatology St Mary's Hospital, Imperial College Healthcare NHS Trust London UK; ^4^ Section of Investigative Medicine and Endocrinology, Department of Metabolism, Digestion and Reproduction, Faculty of Medicine Imperial College London London UK; ^5^ Department of Endocrinology St Mary's Hospital, Imperial College Healthcare NHS Trust London UK; ^6^ MASLD Research Center, Division of Gastroenterology and Hepatology University of California at San Diego La Jolla California USA; ^7^ The George Institute for Global Health (Australia), University of South New Wales Sydney Australia

**Keywords:** ALD, cancers, cardiovascular diseases, fibrosis, MASLD, MetALD, mortality, steatotic liver disease

## Abstract

**Background and Aims:**

We investigated the associations between SLD, fibrosis stage, and all‐cause and cause‐specific mortality, with a focus on SLD subtypes.

**Methods:**

We analysed 486 156 UK Biobank participants. SLD cases were identified using fatty liver index ≥ 60. Causes of death were confirmed via death registries. Multivariable Cox models estimated associations between SLD, SLD subtypes, FIB4 score and mortality outcomes, including all‐cause mortality, mortality from liver‐related diseases, cardiovascular disease (CVD) and extrahepatic cancers.

**Results:**

SLD was identified in 178 336 participants (36.7%): 73.5% with MASLD, 19.0% with MetALD and 6.4% with ALD. Over a median follow‐up of 13.8 years, 20 766 (11.6%) deaths occurred among people with SLD and 21 754 among those without (307 820; 7.1%), suggesting a higher mortality rate in SLD than in non‐SLD (8.78 vs. 5.25/1000 person‐years). All SLD subtypes were associated with higher all‐cause mortality: MASLD (HR (95% CI): 1.32 (1.29–1.35)), MetALD (1.16 (1.12–1.20)) and ALD (1.36 (1.29–1.44)). Excess mortality was primarily driven by extrahepatic cancer (42.5%) and cardiovascular disease (24.2%), while liver‐related deaths were concentrated among those with ALD and fibrosis. A strong dose–response relationship was observed between FIB4 stratification and mortality, particularly for liver‐related deaths. These associations were independent of socioeconomic status, lifestyle and cardiometabolic risk factors.

**Conclusion:**

SLD is independently associated with increased all‐cause and cause‐specific mortality, with substantial variation across subtypes and fibrosis severity. Extrahepatic cancer and cardiovascular disease are the leading contributors to excess mortality. These findings underscore the need for integrated care strategies targeting metabolic risk, fibrosis progression and cancer prevention in the SLD population.

## Lay Summary

In this large population‐based study of nearly half a million adults, 36.7% had SLD, with MASLD being the most common subtype. The leading causes of death in people with SLD were non‐liver cancers and cardiovascular diseases. Having SLD significantly increased the risk of death by 28%, including a 22% higher death risk from cancer, 57% from cardiovascular disease and 136% from liver‐related disease, with risks rising alongside liver fibrosis severity and varying across SLD subtypes.

## 1 Introduction

Steatotic liver disease (SLD) affects one in every three adults worldwide [1]. It encompasses three subtypes: metabolic dysfunction associated steatotic liver disease (MASLD), metabolic dysfunction and alcohol related steatotic liver disease (MetALD) and alcohol related liver disease (ALD). MASLD is considered the liver component of broad metabolic dysfunction [2], and is intricately associated with increased risk of various liver‐related and extrahepatic conditions, such as cirrhosis and hepatocellular carcinoma (HCC), cardiovascular diseases (CVDs) and extrahepatic cancers [3, 4, 5]. MetALD is characterised by both metabolic risk factors and moderate alcohol consumption [6].

While SLD increases hepatic and extrahepatic morbidity, its associations with mortality outcomes are less clearly defined. A 2019 systematic review reported that MASLD increased liver‐related mortality but showed null associations with CVD or cancer mortality [7]. A review in 2023 found positive associations with overall and CVD mortality but not with cancer mortality [8], while another meta‐analysis reported null associations with all‐cause mortality or mortality of liver‐related diseases or HCC but positive associations with extrahepatic cancer and CVD in females [9]. However, these meta‐analyses showed substantial heterogeneity. Mortality risk varies by fibrosis stages among individuals with MASLD [10], with fibrosis widely recognised as the strongest prognostic indicator for adverse outcomes in this population [11]. Non‐invasive assessment of fibrosis has been widely adopted in SLD management [12].

Despite an alarming increase in liver‐related mortality since the 1970s, and the rising burden of SLD in the UK [13], there is a notable lack of UK‐based data on mortality outcomes in this context. Therefore, the objective of this study was to assess causes of death in people with SLD, and to investigate the associations between SLD, fibrosis stage and mortality outcomes, with a focus on the differences between MASLD, MetALD and ALD, in the UK population.

## 2 Methods

### 2.1 Data and Participants

This study utilised data from the UK Biobank, a prospective cohort comprising ~500 000 people aged 40–70 years, recruited between 2006 and 2010. Baseline assessment included collection of data on socioeconomic characteristics, health status, environmental exposure, lifestyle factors, medication use and physical measures. Participants were followed up via linkage to national death registries and hospital records.

SLD was defined as fatty liver index (FLI) ≥ 60. FLI is an indicator for liver steatosis, calculated from body mass index (BMI), waist circumference, triglycerides (TG) and gamma‐glutamyl transferase (GGT) [14]. The cutoff value of 60 to define liver steatosis has been validated and used extensively [15, 16]. SLD subtypes (MASLD, MetALD and ALD) were defined based on alcohol consumption and presence of cardiometabolic risk factors (CMRFs) [17]. The daily alcohol consumption cutoff values for MASLD, MetALD and ALD were < 20/30 (female/male), 20–50/30–60 and > 50/60 g/day, respectively. We assessed the following CMRFs:

- Overweight/obesity (BMI ≥ 25 kg/m^2^ OR waist circumference > 94 cm (male) (> 90 cm (female))),
- prediabetes/diabetes (glycated haemoglobin (HbA1c) ≥ 48 mmol/mol OR diagnosis of type 2 diabetes OR on treatment for type 2 diabetes),
- hypertension (systolic blood pressure (BP) ≥ 130 OR diastolic BP ≥ 85 mmHg OR on antihypertensive drug treatment or diagnosis of hypertension),
- high TG (plasma TG ≥ 1.70 mmol/L OR on lipid lowering treatment), and
- low high‐density lipoprotein (HDL) cholesterol (HDL‐cholesterol ≤ 1.0 mmol/L (male) (≤ 1.3 mmol/L (female)) OR on lipid lowering treatment) [17].

Participants with presence of liver steatosis were categorised as others if they had any of the following chronic liver diseases: viral hepatitis, hemochromatosis, Wilson's disease, biliary cirrhosis, autoimmune hepatitis, primary sclerosing cholangitis, drug‐induced liver injuries, Budd‐Chiari syndrome, etc. These chronic liver diseases were ascertained via self‐report and hospitalisation data; the code lists for these conditions are presented in Appendix S1. We excluded women who were pregnant at baseline and people who had missing data for calculating FLI.

Fibrosis status was measured with FIB4 score [18]. We used the cutoff values 1.30 and 2.67 to categorise low, intermediate and high levels of FIB‐4 score for people < 65 years old, and 2.00 and 3.25 for people ≥ 65 years old [19, 20].

### 2.2 Variable Measurement

The primary outcome of interest was all‐cause mortality, confirmed via death registry records. For cause‐specific mortality, we were interested in deaths due to CVD (ICD10 code: I00‐I99), extrahepatic cancer (C00‐C99, excluding C22) and liver‐related diseases (K70‐K77 for chronic liver disease, C22 for liver cancer). Participants were censored at the date of death or the last date of follow‐up (30 November 2022), whichever occurred first.

Ethnicity was classified into White, Asian, Black and mixed/others. The Townsend Deprivation Index was used to designate socioeconomic status. Lifestyle factors considered were smoking status, alcohol intake and physical activity level. Alcohol intake was assessed via self‐reported consumptions of various alcoholic drinks; the consumptions were summed up to derive average daily alcohol consumption (g/day). Physical activity level was categorised into low, moderate and high levels, based on the frequency, duration and intensity of their physical activities. Systolic and diastolic BP were measured twice and the average values were used. Blood biochemistry markers were measured at a central laboratory, including TG, HDL‐cholesterol, glycated haemoglobin (HbA1c), liver enzymes and haemoglobin levels. For all the categorical covariates, answers of ‘unknown’, ‘do not know’, ‘prefer not to say’ were combined into one ‘unknown’ category.

### 2.3 Statistical Analysis

Cox proportional hazard regression models were used to assess the prospective association between SLD, SLD subtypes, FIB4 score levels and mortality outcomes, expressed as hazard ratio (HR) and 95% confidence interval (CI), using individuals without SLD as the reference group. The models were stratified by region and age group (< vs. ≥ 65 years old). For associations of overall SLD and FIB4 score levels, the models were adjusted for sex, ethnicity, education, Townsend Deprivation Index (in fifths), physical activity level, smoking status and daily alcohol consumption. For the association of SLD subtypes, the model was not adjusted for daily alcohol consumption. We also performed similar analyses to compare the mortality across combinations of SLD subtypes and FIB4 score levels, using people with no SLD as the reference. The proportional hazard assumption was examined by scaled Schoenfeld residuals, and no evidence was observed for its violation. We calculated the p for trend for FIB4 levels, by fitting it as a continuous variable.

We performed several sensitivity analyses to examine the robustness of the findings. First, we additionally adjusted for the CMRFs in Cox models to see if the associations were independent of these risk factors. Second, we removed the first 2 years of follow‐up to correct for reverse causation. Third, we used a more stringent FLI cutoff value (≥ 70) to define liver steatosis. Fourth, we applied an alternative definition of SLD using hepatic steatosis index (HSI) to assess liver steatosis, with HSI > 36 indicating presence of liver steatosis [21]. Although a subset of UK Biobank participants had MRI‐measured liver fat data available [22], which is a more accurate liver fat measure, we could not do a similar sensitivity analysis in this subset, because they did not have FIB4 data measured at liver scans. Fifth, because applying the arbitrary cutoff values for alcohol consumption may introduce misclassification bias into SLD subtypes, we performed a sensitivity analysis removing the participants with borderline alcohol consumption. Borderline alcohol consumption was defined as alcohol intake 2 g/day higher or lower than the cutoff values, which was 28–32 g/day and 58–62 g/day for males, and 18–22 g/day and 48–52 g/day for females, respectively. Sixth, we additionally adjusted for alcohol consumption in the Cox models. Seventh, to account for competing risk, we fitted Fine‐Gray sub‐distribution model to assess the association with cause‐specific mortality. We additionally performed sex‐specific analysis to delineate any sex differences.

All analyses were conducted in R.

## 3 Results

### 3.1 Baseline Characteristics and Fibrosis Burden

**FIGURE 1:**
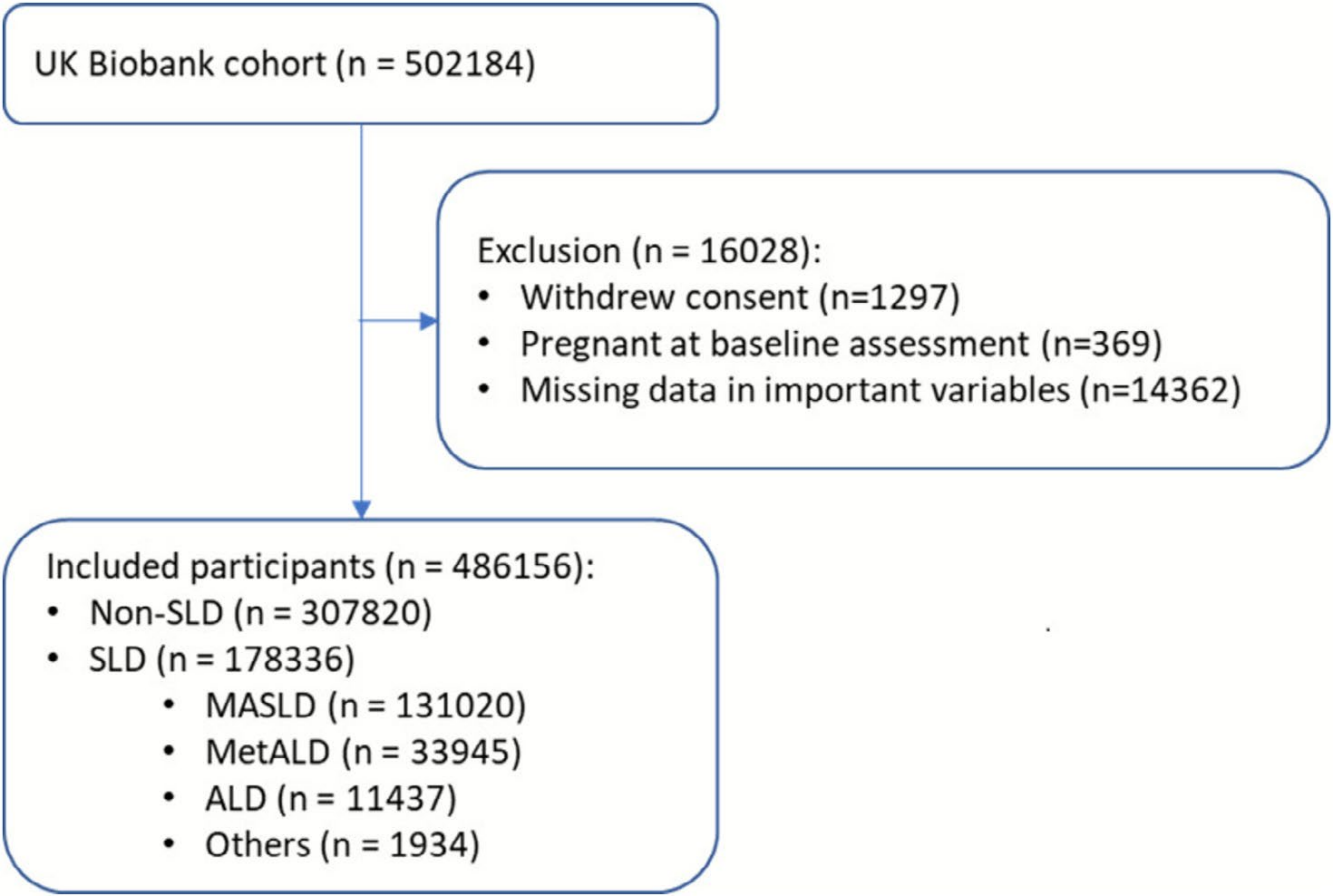
Flowchart of participant selection. ALD, alcohol related liver disease; MASLD, metabolic dysfunction associated steatotic liver disease; MetALD, metabolic dysfunction and alcohol related liver disease; SLD, steatotic liver disease.

Among 486 156 participants, 178 336 (36.7%) were identified with SLD (36.3% females, mean age 57.3 years), comprising 131 020 with MASLD (73.5%), 33 945 with MetALD (19.0%) and 11 437 with ALD (6.4%) (Figure 1).

Compared to individuals without SLD, those with SLD were more likely to be older, male, living in more socioeconomically deprived areas, less educated, less physically active and to have higher alcohol consumption, BMI, blood pressure, TG levels, HbA1c levels and to have lower HDL levels (Table 1, Table S1).

**TABLE 1:** Baseline characteristics of people with and without SLD in the UK Biobank.

	Non‐SLD	SLD				Overall
		All SLD	MASLD	MetALD	ALD	
	*n* = 307 820	*n* = 178 336	*n* = 131 020	*n* = 33 945	*n* = 11 437	*n* = 486 156
Sex, male	108 955 (35.4%)	113 587 (63.7%)	76 275 (58.2%)	25 701 (75.7%)	10 312 (90.2%)	222 542 (45.8%)
Age, years	56.1 (8.2)	57.3 (7.8)	57.4 (7.9)	57.0 (7.7)	56.3 (7.7)	56.6 (8.1)
Townsend deprivation index						
1st fifth (least deprived)	65 121 (21.2%)	32 462 (18.2%)	23 791 (18.2%)	6619 (19.5%)	1799 (15.7%)	97 583 (20.1%)
5th fifth (most deprived)	56 140 (18.2%)	40 870 (22.9%)	30 641 (23.4%)	6584 (19.4%)	2950 (25.8%)	97 010 (20.0%)
Education, higher education	109 872 (35.7%)	47 346 (26.5%)	34 208 (26.1%)	9794 (28.9%)	2811 (24.6%)	157 218 (32.3%)
Ethnicity, White	290 659 (94.4%)	168 183 (94.3%)	121 919 (93.1%)	33 225 (97.9%)	11 215 (98.1%)	458 842 (94.4%)
Smoking, never	179 488 (58.3%)	85 523 (48%)	69 146 (52.8%)	12 397 (36.5%)	3232 (28.3%)	265 011 (54.5%)
Alcohol drinking, g/d^a^	9.7 (0.7, 20.0)	11.9 (0.0, 28.6)	5.7 (0.0, 14.8)	37.9 (32.2, 45.9)	75.4 (65.8, 91.6)	10.2 (0.4, 22.4)
Physical activity, high	106 203 (34.5%)	47 835 (26.8%)	33 406 (25.5%)	10 236 (30.2%)	3703 (32.4%)	154 038 (31.7%)
Hypertension	188 397 (61.2%)	145 417 (81.5%)	105 177 (80.3%)	28 663 (84.4%)	10 115 (88.4%)	333 814 (68.7%)
Overweight/obesity	172 114 (55.9%)	175 794 (98.6%)	129 553 (98.9%)	33 341 (98.2%)	11 058 (96.7%)	347 908 (71.6%)
Prediabetes/Diabetes	38 433 (12.5%)	51 762 (29.0%)	40 909 (31.2%)	7641 (22.5%)	2595 (22.7%)	90 195 (18.6%)
High triglycerides	77 920 (25.3%)	128 292 (71.9%)	95 116 (72.6%)	23 904 (70.4%)	8057 (70.4%)	206 212 (42.4%)
Low HDL‐cholesterol	92 556 (30.1%)	73 177 (41.0%)	61 157 (46.7%)	9109 (26.8%)	2117 (18.5%)	165 733 (34.1%)

*Note:* The cutoff values for low, intermediate and high FIB4 scores were 1.30 and 2.67 for people < 65 years old, and 2.00 and 2.67 for people > = 65 years old.

Abbreviations: HbA1c, glycated haemoglobin; HDL, high density lipoprotein.

^a^ Showing median (interquartile interval).

**FIGURE 2:**
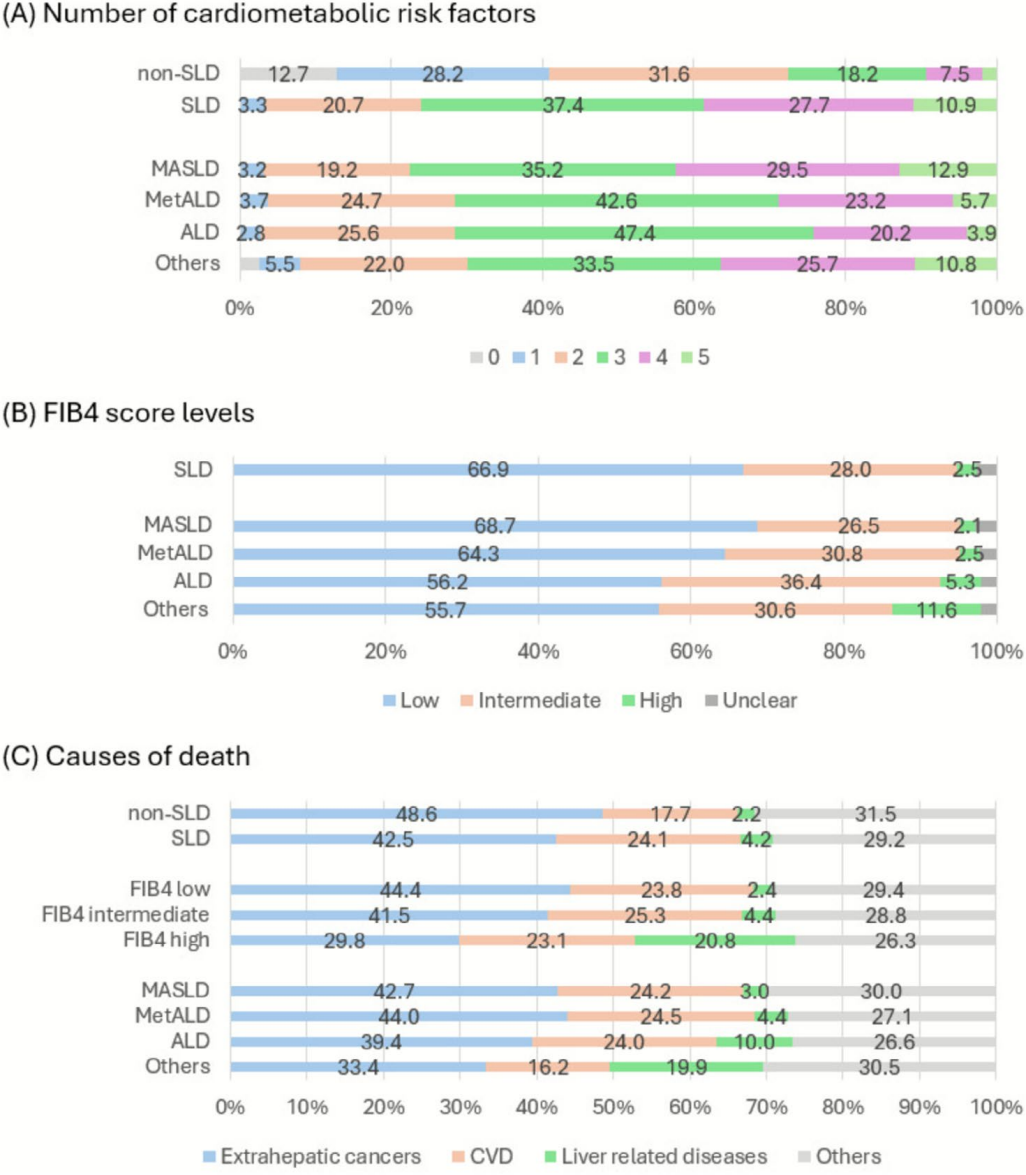
Distributions of FIB4 score levels, number of cardiometabolic risk factors and causes of death in people with and without SLD. The numbers show the percentage. The cutoff values for low, intermediate and high FIB4 scores were 1.30 and 2.67 for people < 65 years old, and 2.00 and 2.67 for people > = 65 years old. SLD, steatotic liver disease.

The burden of CMRFs was highest among individuals with MASLD, with 12.9% of individuals having five CMRFs, compared to 5.7% in MetALD and 3.9% in ALD (Figure 2A). In MASLD, 98.9% were classified as overweight/obese, 80.3% hypertensive and 72.6% high TG. In contrast, MetALD and ALD participants exhibited higher levels of alcohol intake and smoking. Among non‐SLD individuals, the three most prevalent CMRFs were hypertension (61.2%), overweight/obesity (55.9%) and low HDL‐cholesterol (30.1%).

FIB4 scores were calculable in 173 711 (97.4%) of SLD participants: 66.9% had low FIB4, and 2.5% high. MASLD had the highest proportion of low FIB4 scores (68.7%), whereas ALD had the highest proportion of high FIB4 scores (5.3%) (Figure 2B).

### 3.2 Mortality in SLD Versus Non‐SLD

Over a median follow‐up of 13.8 years, 20 766 deaths occurred among the SLD group and 21 754 among non‐SLD (mortality rate: 8.78 vs. 5.25/1000 person‐years). Extrahepatic cancer and CVD were the leading causes of death in both groups. Among non‐SLD people, extrahepatic cancer accounted for 48.6% of deaths, followed by CVD (17.7%) and liver‐related diseases (2.2%). In people with SLD, extrahepatic cancer remained the leading cause (42.5%), but the proportions of CVD (24.2%) and liver‐related deaths (4.2%) were higher than in the non‐SLD group (Figure 2C).

SLD was associated with elevated mortality risks compared to non‐SLD, including all‐cause mortality (HR (95% CI): 1.28 (1.26, 1.31)), extrahepatic cancer mortality (1.22 (1.19, 1.26)), CVD mortality (1.57 (1.50, 1.64)) and liver‐related disease mortality (2.36 (2.09, 2.65)) (Table 2).

**TABLE 2:** Adjusted associations between SLD, FIB4 scores and all‐cause and selected disease‐specific mortality.

	Non‐SLD	SLD				
		All SLD	Low FIB4	Intermediate FIB4	High FIB4	*p* for trend
	*n* = 307 820	*n* = 178 336	*n* = 119 362	*n* = 49 888	*n* = 4461	
All‐causes combined						
Death, *n*	21 754	20 766	12 589	6271	1334	
Mortality rate/1000py	5.25	8.78	7.89	9.57	25.01	
HR (95% CI)	Reference	1.28 (1.26, 1.31)	1.11 (1.09, 1.14)	1.63 (1.58, 1.68)	2.62 (2.48, 2.77)	< 0.01
Extrahepatic cancer						
Death, *n*	10 573	8819	5588	2604	398	
Mortality rate/1000py	2.55	3.73	3.5	3.98	7.46	
HR (95% CI)	Reference	1.22 (1.19, 1.26)	1.11 (1.07, 1.15)	1.49 (1.42, 1.56)	1.84 (1.66, 2.04)	< 0.01
CVD						
Death, *n*	3860	4997	2977	1575	306	
Mortality rate, /1000py	0.93	2.11	1.87	2.4	5.74	
HR (95% CI)	Reference	1.57 (1.50, 1.64)	1.34 (1.28, 1.41)	2.08 (1.96, 2.22)	2.88 (2.56, 3.25)	< 0.01
Liver‐related diseases						
Death, *n*	472	874	300	276	277	
Mortality rate/1000py	0.11	0.37	0.19	0.42	5.19	
HR (95% CI)	Reference	2.36 (2.09, 2.65)	1.25 (1.07, 1.45)	2.91 (2.49, 3.40)	29.23 (24.86, 34.37)	< 0.01

*Note:* Model was stratified by region and age group (< 65 vs. > = 65) and adjusted for sex, ethnicity, education, Townsend Deprivation Index, physical activity level, smoking, drinking. The cutoff values for low, intermediate and high FIB4 scores were 1.30 and 2.67 for people < 65 years old, and 2.00 and 2.67 for people > = 65 years old.

Abbreviations: CVD, cardiovascular disease; HCC, hepatocellular carcinoma; HR (95% CI), hazard ratio (95% confidence interval); py, person‐years; SLD, steatotic liver disease.

A strong dose–response relationship was observed between FIB4 score levels and mortality outcomes. For example, compared to non‐SLD, individuals with low, intermediate and high FIB4 levels had 11% (1.11 (1.09, 1.14)), 63% (1.63 (1.58, 1.68)) and 162% (2.62 (2.48, 2.77)) increased risks of all‐cause mortality, respectively. The association was most pronounced for liver‐related mortality, with according HR estimates of 1.25 (1.07, 1.45), 2.91 (2.49, 1.45) and 29.23 (24.86, 34.37) (Table 2).

### 3.3 MASLD

**FIGURE 3:**
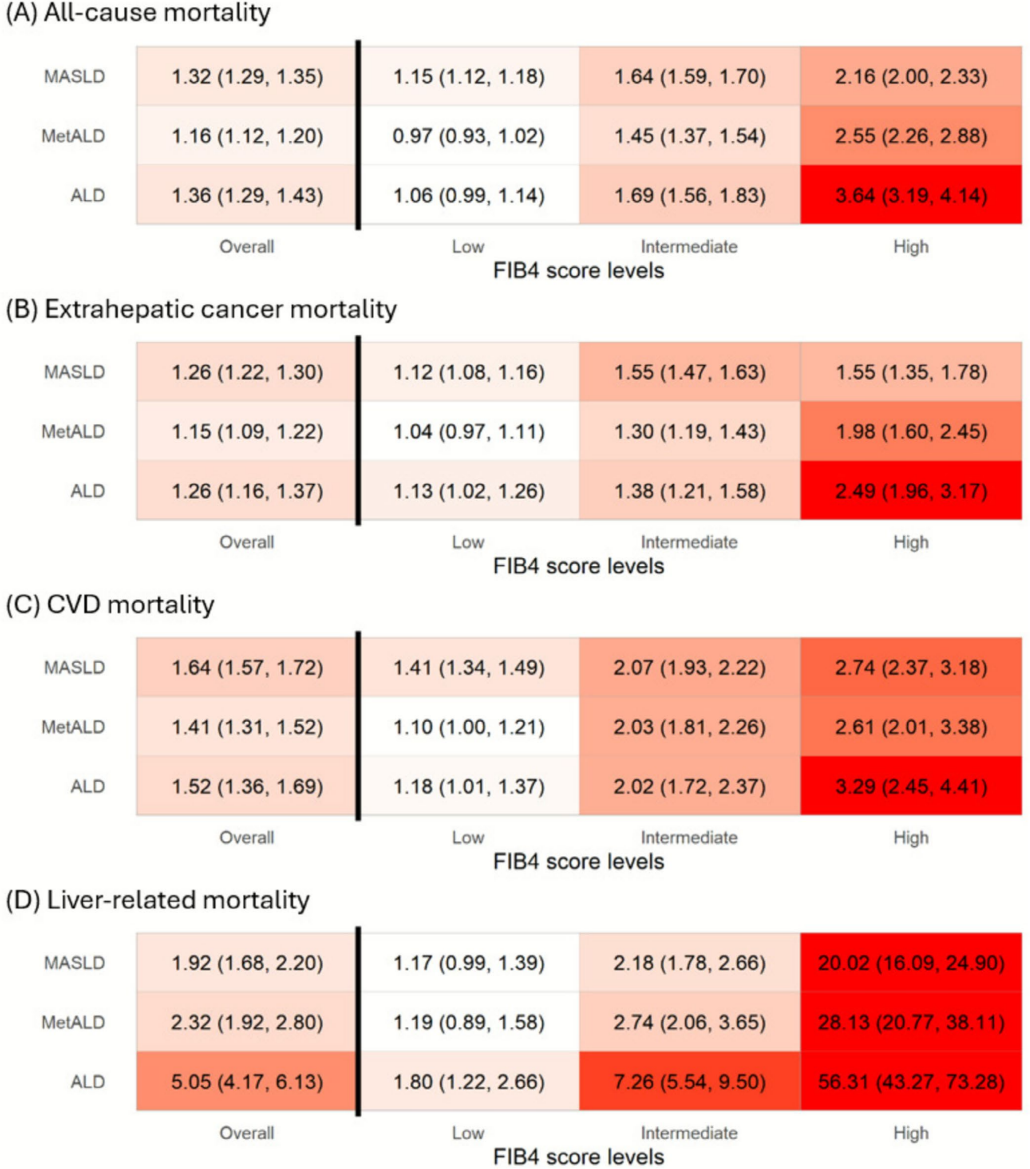
Heatmap of associations between SLD subtypes and all‐cause and cause‐specific mortality, stratified by FIB4 scores. (A) All cause mortality. (B) Extrahepatic cancer mortality. (C) CVD mortality. (D) Liver‐related mortality. ALD, alcohol related liver disease; MASLD, metabolic dysfunction associated steatotic liver disease; MetALD, metabolic dysfunction and alcohol related liver disease; SLD, steatotic liver disease. The cutoff values for low, intermediate and high FIB4 scores were 1.30 and 2.67 for people < 65 years old, and 2.00 and 2.67 for people > = 65 years old. Mortality rate: Per 1000 person‐years. The Cox was stratified by age group (< 65 vs. > = 65 years) and regions, and adjusted for sex, ethnicity, Townsend Deprivation Index, education, smoking and physical activity. The colour represents HR estimates.

Individuals with MASLD had the highest cardiovascular burden, with 77.6% presenting with ≥ 3 CMRFs. Despite this, they had the most favourable fibrosis profile, with 68.7% exhibiting low FIB4 scores and only 2.1% high FIB4 scores. During follow‐up, extrahepatic cancer was the leading cause of death (42.7%), followed by CVD (24.2%) and liver‐related causes (3.0%) (Figure 2C). Compared to non‐SLD individuals, MASLD was associated with elevated risks of all‐cause mortality (HR 1.32 (1.29, 1.35)), extrahepatic cancer mortality (1.26 (1.22, 1.30)), CVD mortality (1.64 (1.57, 1.73)) and liver‐related mortality (1.92 (1.68, 2.20)) (Figure 3).

FIB4‐stratified analyses showed a clear dose–response relationship for all‐cause mortality, with HR estimates of 1.15 (1.12, 1.18), 1.64 (1.59, 1.70) and 2.16 (2.00, 2.33) for low, intermediate and high FIB4 scores, respectively. Similar trends were observed for cause‐specific mortality, with high FIB4 scores associated with a markedly increased risk of liver‐related death (20.02 (16.09, 24.90)) (Figure 3).

### 3.4 MetALD

Participants with MetALD displayed a mixed risk profile with both cardiometabolic and alcohol‐related factors. Their fibrosis burden was slightly more advanced than MASLD, with 64.3% with low FIB4 and 2.5% with high FIB4. The leading causes of death were extrahepatic cancer (44.0%) and CVD (24.5%), and 4.4% of deaths were due to liver‐related diseases.

Compared to non‐SLD, MetALD was associated with increased all‐cause mortality (1.16 (1.12–1.20)), extrahepatic cancer mortality (1.15 (1.09–1.22)), CVD mortality (1.41 (1.31–1.52)) and liver‐related mortality (2.32 (1.92–2.80)).

FIB4 stratification again revealed a dose–response pattern: HR estimates for all‐cause mortality were 0.97 (0.93–1.02), 1.45 (1.37, 1.54) and 2.55 (2.26, 2.88) for low, intermediate and high FIB4 score levels. High FIB4 scores were associated with a sharp increase in liver‐related mortality (28.13 (20.77, 38.11)).

### 3.5 ALD

ALD participants had the most advanced fibrosis burden and the lowest prevalence of cardiometabolic comorbidities. Only 56.2% had low FIB4 scores, while 5.3% had high scores. This group exhibited the highest alcohol intake (mean (SD): 83.8 (27.9) g/day) and current smoking rate (21.7%).

Although extrahepatic cancer and CVD still dominated causes of death (39.4% and 24.0%, respectively) in ALD, liver‐related death accounted for 10.0% of mortality, notably higher than in people with MASLD or MetALD.

ALD was associated with increased mortality risks, with HR 1.36 (1.29, 1.44) for all‐cause mortality, 1.26 (1.16, 1.37) for extrahepatic cancer mortality, 1.52 (1.36, 1.69) for CVD mortality and 5.05 (4.17–6.13) for liver‐related mortality, respectively.

FIB4 scores were strongly associated with mortality outcomes. For all‐cause mortality, the HRs were 1.06 (0.99, 1.14), 1.69 (1.56, 1.83) and 3.64 (3.19, 4.14) for low, intermediate and high FIB4 score levels, respectively. High FIB4 score was strongly associated with liver‐related mortality, with HRs of 7.26 (5.54, 9.50) and 56.31 (43.27, 73.26) for intermediate and high FIB4 scores (Figure 3).

### 3.6 Sensitivity and Sex‐Stratified Analyses

All associations remained robust after additionally adjusting for CMRFs, indicating that SLD was an independent risk factor. Excluding the first 2 years of follow‐up yielded consistent results, reducing the likelihood of reverse causation bias (Tables S2 and S3).

Sensitivity analysis using HSI to define liver steatosis yielded consistent findings (Table S4). Among all participants with available data on FLI and HSI, the FLI definition identified 178 336 participants with SLD, while the HSI definition identified 211 097 SLD cases, including 163 467 (77.4%) MASLD, 35 850 MetALD (17.0%) and 9359 (4.4%) ALD. When examining the association between SLD subtypes and all‐cause mortality, the HR estimates were 1.23 (1.20, 1.26), 1.07 (1.03, 1.11) and 1.22 (1.14, 1.29) for MASLD, MetALD and ALD, respectively. These findings closely mirror the primary analyses using FLI to define SLD, reinforcing the robustness of our findings across different case definitions.

Sensitivity analysis using a more stringent FLI cutoff value (> 70) for liver steatosis also showed consistent results to the primary analysis (Table S5). Consistent results were also observed in sensitivity analysis excluding 32 612 participants with borderline alcohol consumption (Table S6), and sensitivity analysis with additional adjustment of alcohol consumption (Table S7).

To account for competing risk, we fitted Fine‐Gray models to assess the associations between SLD subtypes and mortality from extrahepatic cancer and CVD, showing consistent results with the primary analysis. For mortality from extrahepatic cancer, the HR estimates were 1.25 (1.21, 1.29), 1.15 (1.08, 1.22) and 1.24 (1.15, 1.34) for MASLD, MetALD and ALD, respectively. For mortality from CVD, the according HR estimates were 1.64 (1.57, 1.72), 1.41 (1.31, 1.51) and 1.48 (1.33, 1.66) (Table S8). We did not fit the Fine‐Gray model for associations with mortality from liver‐related diseases, due to their small number of events, in which case Fine‐Gray model results may be unstable.

Sex‐stratified analyses showed stronger associations in females than in males. For instance, SLD versus non‐SLD HRs for all‐cause mortality were 1.45 (1.40, 1.50) in females and 1.18 (1.15, 1.21) in males. This pattern was consistent for extrahepatic cancer mortality (1.33 (1.27, 1.39) vs. 1.15 (1.10, 1.19)), CVD mortality (1.86 (1.72, 2.01) vs. 1.43 (1.36, 1.51)) and liver‐related mortality (2.68 (2.23, 3.48) vs. 2.17 (1.87, 2.53)) (Tables S2 and S9).

## 4 Discussion

Our findings underscore the high prevalence and serious adverse prognosis of SLD. In this study of over 486 000 middle‐aged participants, one in three was classified as SLD, in line with the global prevalence [1].

Crucially, extrahepatic causes dominate the mortality spectrum in SLD. In our cohort, ≤ 5% of deaths were due to liver‐related diseases, whereas 67.6% were attributable to extrahepatic cancer and CVD, which was consistent with some previous findings [23]. For example, a NHANES study found that CVD and neoplasms were the leading causes of death in people with MASLD, followed by liver‐related diseases [24]. In a Swedish study [25], extrahepatic cancers represented the most common causes of death in people with MASLD.

However, the actual composition of causes of death varied across studies, mainly influenced by SLD disease severity. For example, a US study [26], including 44 888 individuals with NAFLD from the US National Vital Statistics System, reported liver‐related diseases were the most common cause (45.8%), while CVD (11.4%) and extrahepatic cancer (7.0%) together accounted for only 18.4%, and that liver cancers represented the highest proportion (41.0%) among all cancers. This was likely due to their use of ICD10 codes to define NAFLD cases, which preferentially identified more advanced liver disease (80% with cirrhosis). A prospective cohort of 1773 adults with NAFLD (75% with steatohepatitis, 30% with fibrosis or cirrhosis) similarly found that about 25% of total deaths were attributed to liver‐related diseases [27]. Compared to these studies, our study found an overall substantially lower liver‐related mortality, because the UK Biobank was based on the general population, instead of on clinical populations or administrative databases, which are more likely to capture advanced diseases. Furthermore, in our study, SLD was defined using FLI, instead of clinical diagnosis of ICD10 codes, which would also presumably identify more severe cases. Having said that, these variations actually align with our findings that the distribution of causes of death changes across FIB4 score levels, with higher proportions of liver‐related disease in the high FIB4 group.

We observed that SLD was associated with increased overall and cause‐specific mortality, which remained evident after accounting for demographic, lifestyle and cardiometabolic factors as well as reverse causation bias, suggesting that SLD itself contributes to worse outcomes.

Associations between SLD, its severity and mortality from cancer and CVD have been substantially studied, although inconsistencies remain [7, 8, 9]. A Swedish study showed that MASLD and its severity are positively linked to mortality of cancer, CVD and cirrhosis, and the excess mortality was mainly due to extrahepatic cancers [25], which was similar to our findings. This study also reported cirrhosis was the second largest contributor to excess mortality, outranking CVD, which could be due to the higher proportion of advanced fibrosis and cirrhosis in their cohort (21.3%) compared to ours (1.5%). Yi et al. [28] and Mantovani et al. [29] pointed out that the heterogeneity across studies is attributed to variations in populations, SLD diagnosis criteria, follow‐up length and covariate adjustment.

Our findings also confirmed that liver fibrosis remains a crucial prognostic driver within SLD. Mortality risk increased progressively with higher FIB4 scores, showing a clear dose–response relationship. This stepwise risk aligns with earlier evidence that fibrosis stage is the strongest predictor of outcomes in liver diseases [10, 11, 30].

We found the associations with cause‐specific mortality varied across SLD subtypes. For example, the HR estimates increased progressively from MASLD to MetALD and ALD for liver‐related mortality, consistent with the findings from Israelsen et al. in a Danish study investigating the risk of liver decomposition [31]. However, for all‐cause mortality, we found MASLD and ALD showed similar HRs, higher than MetALD, whereas Israelsen et al. reported a progressive increase in risk from MASLD to MetALD and ALD. This discrepancy may be due to the different disease severity between the two cohorts; in our study only 2.6% of patients had high FIB4 scores, compared with 59% of participants with significant fibrosis and 18% with cirrhosis in the Danish cohort. Notably, when we restricted analyses to individuals with high FIB4 scores, we observed a progressive increase of HR estimates from MASLD to MetALD and ALD, similar to the Danish cohort. We also observed that people with MetALD were more likely to have higher socioeconomic status and educational attainment, which may contribute to a slightly reduced mortality compared to MASLD and ALD. However, we acknowledge that MetALD is a heterogeneous subtype with varying degrees of cardiometabolic burden and alcohol‐related liver injury, making it difficult to understand its true disease burdens and to compare across studies, as the burden of cardiometabolic risk factors and alcohol intake may vary across populations and studies. Interestingly, for CVD mortality, MASLD was associated with a higher HR than MetALD and ALD, indicating a strong contribution of cardiometabolic burden to CVD mortality [32], as individuals with MASLD tended to have a higher cardiometabolic burden. In our data, 42.4% of MASLD participants had 4+ CMRFs, substantially higher than MetALD (28.9%) and ALD (24.1%).

The mechanisms linking SLD, CVD and extrahepatic cancers have been widely discussed [5, 33, 34]. These conditions share common CMRFs. However, our sensitivity analysis showed the association was independent of CMRF. The ‘parallel hits’ hypothesis highlighted four key pathophysiological mechanisms: lipotoxicity, insulin resistance, proinflammatory diets and intestinal dysbiosis, which collectively contribute to liver and systemic inflammation. Chronic liver inflammation promotes liver fibrosis and the eventual progression towards cirrhosis and HCC. The low‐grade systemic inflammation further contributes to the development of cardiovascular inflammation, atherosclerosis and chronic kidney disease [5, 33]. Additionally, chronic inflammation facilitates tumourigenesis with NF‐kB activation as a crucial driver. Other mechanisms also include adipokine signalling disturbance caused by obesity, increased production of reactive oxygen species, insulin‐like growth factor‐1 and insulin resistance via activation of other inflammatory pathways [5, 34].

We observed that the SLD‐associated increased mortality was generally stronger in females than in males. Similar patterns have been reported in previous studies; for example, Ji et al. found a stronger association with all‐cause mortality in females than in males [35], while Liu et al. [7] and Yi et al. [28] reported stronger associations with overall mortality and CVD mortality in females, though not statistically significant. Other studies have also observed sex differences in other outcomes, such as cancer and CVD incidences [9, 36, 37]. The observed sex dimorphism may reflect a combination of biological and lifestyle factors, including the influence of sex hormones, differences in adipose tissue distribution and lipotoxicity, alterations in the gut‐liver axis and variations in skeletal muscle mass and insulin sensitivity, all of which may further contribute to liver inflammation and fibrosis [34, 38, 39, 40]. Future research is warranted to better elucidate the mechanisms underlying sex differences in SLD [40, 41].

Given that extrahepatic cancer and CVD are the leading causes of death in people with SLD, particularly in those with early disease stage, management strategies should target these conditions to reduce overall mortality and consider sex differences, whilst preventing fibrosis progression using non‐invasive tests, such as FIB4, remains critical.

For extrahepatic cancer, several SLD‐related cancers (e.g., colorectal cancer, breast cancer, prostate cancer, etc.) could be identified by screening [34], however, research evidence is warranted to examine whether the elevated risks require intensive cancer screening in the SLD population and how.

Comprehensive CVD risk assessment is important for disease management, as both SLD and fibrosis are independent risk factors for CVD incidence and mortality [42, 43]. However, current CVD risk prediction models do not incorporate these factors [5], underscoring a need for model updates. Guidelines emphasise lifestyle modifications, including exercise and dietary changes [44], which not only address SLD but also offer significant benefits for cardiometabolic health.

This study has several strengths, including its large sample size, long follow‐up duration and a comprehensive follow‐up via death registries. However, we acknowledge some limitations. First, we used FLI for liver steatosis. Although FLI has been validated and commonly used in previous research [45], and our sensitivity analyses of using a different FLI cutoff value and HSI generated consistent results, it is not an accurate measure of liver steatosis and misclassification bias may still exist, particularly in borderline cases. Future research employing more accurate measures for liver steatosis is warranted. Second, we used FIB4 score for liver fibrosis and age‐specific cutoff values for fibrosis severity levels. Although widely used, this may still introduce measurement bias. The observed HR for high FIB4 and mortality outcomes, especially liver‐related mortality, seems substantially higher than low or intermediate FIB4 levels. Although the directionality was consistent with previous studies, the association magnitude may be partly caused by small event number and misclassification. Therefore, our results should be interpreted with caution. Third, using self‐reported alcohol intake and arbitrary cutoff values may not capture the complexity of real‐world drinking patterns, thus introducing measurement and misclassification biases. Although we performed sensitivity analysis excluding participants with borderline alcohol intake and found consistent results, biases still prevail. Future studies should employ more accurate measurement of alcohol intake. Fourth, UK Biobank is not fully representative of the general UK population in terms of demography, ethnicity, socioeconomic status and disease prevalence [46]. Although comparing to the National Registries in England and Wales [47], our results align with the general observation that cancers and CVD are the most common causes of death in the UK, our findings should be interpreted with caution regarding generalizability to other populations. The small number of liver‐related deaths reflected the health volunteer bias inherent in the cohort and relatively early disease stage caused by case identification method. This would impact the generalizability of our results to clinical populations where SLD patients typically present with more advanced disease. It also makes it difficult to examine any meaningful subgroup differences, and may undermine the general use of UK Biobank for liver‐related research. Fifth, we lacked longitudinal data on SLD progression, which may impact mortality. It is estimated that only 20% of liver steatosis would progress to steatohepatitis, and 20% of steatohepatitis would progress to cirrhosis over 30–40 years [48], suggesting that SLD status likely remained stable over time for most participants. However, future studies with individual level data on SLD progression (such as repeat imaging or biomarker assessment) are warranted.

## 5 Conclusion

In this large prospective cohort study of nearly half a million participants, SLD was associated with increased all‐cause and cause‐specific mortality, with extrahepatic cancers and CVD being the leading contributors to excess mortality. The elevated mortality was independent of CMRF and consistent across fibrosis stages and SLD subtypes. MASLD, MetALD and ALD demonstrated different patterns in increasing mortality. These findings highlight the broad systemic impact of SLD beyond liver‐related complications and underscore the need for a comprehensive, multidisciplinary management approach that integrates cardiometabolic risk reduction, cancer prevention and fibrosis mitigation.

## Author Contributions

Q.F. conceived the research idea, conducted data analysis, and drafted the manuscript. All authors interpreted results, critically reviewed and revised the manuscript.

## Funding

This research/study/project was funded/supported by the NIHR Imperial Biomedical Research Centre (BRC) [NIHR203323]. The views expressed are those of the author(s) and not necessarily those of the NIHR or the Department of Health and Social Care. The Section of Investigative Medicine and Endocrinology at Imperial College London is funded by grants from the MRC, NIHR and is supported by the NIHR Biomedical Research Centre Funding Scheme and the NIHR/Imperial Clinical Research Facility. CI is funded by an NIHR Senior Clinical and Practitioner Research Award (NIHR304591) and an NIHR Imperial BRC Pilot Grant (PSR328). The Division of Digestive Diseases at Imperial College London receives financial support from the National Institute of Health Research (NIHR), Imperial Biomedical Research Centre (BRC) based at Imperial College London and Imperial College Healthcare NHS Trust.

## Conflicts of Interest

C.N.I.‐E. has conducted consultancy work for Novo Nordisk outside the submitted work. All the other authors have nothing to declare.

## Supporting information


**Appendix S1:** liv70485‐sup‐0001‐AppendixS1.docx.

## Data Availability

UK Biobank data are available to registered researchers at https://www.ukbiobank.ac.uk/. Analytic codes are available upon reasonable request.
