# Supplementary material for: Unveiling the Burden of Steatotic Liver Disease: Mortality Risks by Subtype and Fibrosis Stage in a Nationwide Cohort
Source: Liver Int. 2025 Dec 29;46(2):e70485. doi: 10.1111/liv.70485 (PMC12747130; doi:10.1111/liv.70485)
Supplement: Supplementary file 1 — Appendix S1: liv70485‐sup‐0001‐AppendixS1.docx. [file LIV-46-0-s001.docx]

Unveiling the Burden of Steatotic Liver Disease: Mortality Risks by Subtype and Fibrosis Stage in a Nationwide Cohort

Supplementary materials

Table of Contents

[Supplementary methods 2](#_Toc210992517)

[Supplementary table 1: baseline characteristics of people with and without SLD in the UK Biobank 3](#_Toc210992518)

[Supplementary table 2: association between SLD, FIB4 score levels and all-cause and disease-specific mortality, stratified by sex 6](#_Toc210992519)

[Supplementary table 3: sensitivity analyses for adjusted associations between SLD subtypes, FIB4 scores and all-cause and selected disease-specific mortality. 10](#_Toc210992520)

[Supplementary table 4: Adjusted associations between HSI-defined SLD subtypes, FIB4 scores, and all-cause and cause-specific mortality. 12](#_Toc210992521)

[Supplementary table 5: Adjusted associations between SLD subtypes, FIB4 scores, and all-cause and cause-specific mortality using fatty liver index > 70 for liver steatosis. 13](#_Toc210992522)

[Supplementary table 6: Adjusted associations between SLD subtypes, FIB4 scores, and all-cause and cause-specific mortality excluding participants with borderline alcohol consumption. 14](#_Toc210992523)

[Supplementary table 7: Adjusted associations between SLD subtypes, FIB4 scores, and all-cause and cause-specific mortality with additional adjustment of alcohol intake. 15](#_Toc210992524)

[Supplementary table 8: Adjusted associations between SLD subtypes, and cause-specific mortality estimated with Fine-Gray model. 16](#_Toc210992525)

[Supplementary table 9: Adjusted associations between SLD subtypes, FIB4 scores and all-cause and selected disease-specific mortality in males and females. 17](#_Toc210992526)

### Supplementary methods

The codelists for chronic liver diseases can be found online: [codelists.xlsx](https://docs.google.com/spreadsheets/d/1pErQT3krBj3ZpVk2JhM5styq_MYxzQOl/edit?usp=sharing&ouid=108654502587298592146&rtpof=true&sd=true)

Link to the codelist file: <https://docs.google.com/spreadsheets/d/1pErQT3krBj3ZpVk2JhM5styq_MYxzQOl/edit?usp=sharing&ouid=108654502587298592146&rtpof=true&sd=true>

### Supplementary table 1: baseline characteristics of people with and without SLD in the UK Biobank

|  |  | SLD | | | |  |
| --- | --- | --- | --- | --- | --- | --- |
|  | Non-SLD | All SLD | MASLD | MetALD | ALD | Overall |
|  | n = 307820 | n = 178336 | n = 131020 | n = 33945 | n = 11437 | n = 486156 |
| **Sex, male** | 108955 (35.4%) | 113587 (63.7%) | 76275 (58.2%) | 25701 (75.7%) | 10312 (90.2%) | 222542 (45.8%) |
| **Age, years** | 56.1 (8.2) | 57.3 (7.8) | 57.4 (7.9) | 57.0 (7.7) | 56.3 (7.7) | 56.6 (8.1) |
| **Townsend deprivation index** |  |  |  |  |  |  |
| 1st fifth (least deprived) | 65121 (21.2%) | 32462 (18.2%) | 23791 (18.2%) | 6619 (19.5%) | 1799 (15.7%) | 97583 (20.1%) |
| 2nd fifth | 63441 (20.6%) | 33724 (18.9%) | 24624 (18.8%) | 6863 (20.2%) | 1961 (17.1%) | 97165 (20%) |
| 3rd fifth | 62436 (20.3%) | 34475 (19.3%) | 25204 (19.2%) | 6804 (20%) | 2171 (19%) | 96911 (19.9%) |
| 4th fifth | 60316 (19.6%) | 36584 (20.5%) | 26593 (20.3%) | 7047 (20.8%) | 2536 (22.2%) | 96900 (19.9%) |
| 5th fifth (most deprived) | 56140 (18.2%) | 40870 (22.9%) | 30641 (23.4%) | 6584 (19.4%) | 2950 (25.8%) | 97010 (20%) |
| Missing | 366 (0.1%) | 221 (0.1%) | 167 (0.1%) | 28 (0.1%) | 20 (0.2%) | 587 (0.1%) |
| **Education** |  |  |  |  |  |  |
| Below secondary | 48126 (15.6%) | 40157 (22.5%) | 30779 (23.5%) | 6412 (18.9%) | 2440 (21.3%) | 88283 (18.2%) |
| Lower secondary | 52108 (16.9%) | 29186 (16.4%) | 21283 (16.2%) | 5586 (16.5%) | 2020 (17.7%) | 81294 (16.7%) |
| Higher secondary | 17567 (5.7%) | 9050 (5.1%) | 6453 (4.9%) | 1830 (5.4%) | 658 (5.8%) | 26617 (5.5%) |
| Vocational | 80078 (26%) | 52492 (29.4%) | 38195 (29.2%) | 10323 (30.4%) | 3508 (30.7%) | 132570 (27.3%) |
| Higher education | 109872 (35.7%) | 47346 (26.5%) | 34208 (26.1%) | 9794 (28.9%) | 2811 (24.6%) | 157218 (32.3%) |
| Missing | 69 (0%) | 105 (0.1%) | 102 (0.1%) | 0 (0%) | 0 (0%) | 174 (0%) |
| **Ethnicity** |  |  |  |  |  |  |
| White | 290659 (94.4%) | 168183 (94.3%) | 121919 (93.1%) | 33225 (97.9%) | 11215 (98.1%) | 458842 (94.4%) |
| Asian | 6934 (2.3%) | 3849 (2.2%) | 3536 (2.7%) | 216 (0.6%) | 63 (0.6%) | 10783 (2.2%) |
| Black | 4553 (1.5%) | 2944 (1.7%) | 2682 (2.0%) | 188 (0.6%) | 43 (0.4%) | 7497 (1.5%) |
| Others | 4560 (1.5%) | 2541 (1.4%) | 2212 (1.7%) | 219 (0.6%) | 74 (0.6%) | 7101 (1.5%) |
| Missing | 1114 (0.4%) | 819 (0.5%) | 671 (0.5%) | 97 (0.3%) | 42 (0.4%) | 1933 (0.4%) |
| **Smoking** |  |  |  |  |  |  |
| Never | 179488 (58.3%) | 85523 (48%) | 69146 (52.8%) | 12397 (36.5%) | 3232 (28.3%) | 265011 (54.5%) |
| Previous | 96646 (31.4%) | 71395 (40%) | 48166 (36.8%) | 16729 (49.3%) | 5698 (49.8%) | 168041 (34.6%) |
| Current | 30546 (9.9%) | 20438 (11.5%) | 12906 (9.9%) | 4675 (13.8%) | 2482 (21.7%) | 50984 (10.5%) |
| Missing | 1140 (0.4%) | 980 (0.5%) | 802 (0.6%) | 144 (0.4%) | 25 (0.2%) | 2120 (0.4%) |
| **Alcohol drinking, g/day** | 9.7 (0.7, 20.0) | 11.9 (0.0, 28.6) | 5.7 (0.0, 14.8) | 37.9 (32.2, 45.9) | 75.4 (65.8, 91.6) | 10.2 (0.4, 22.4) |
| **Physical activity** |  |  |  |  |  |  |
| Low | 36890 (12%) | 32501 (18.2%) | 24176 (18.5%) | 5883 (17.3%) | 2085 (18.2%) | 69391 (14.3%) |
| Moderate | 98185 (31.9%) | 54899 (30.8%) | 39507 (30.2%) | 11194 (33%) | 3578 (31.3%) | 153084 (31.5%) |
| High | 106203 (34.5%) | 47835 (26.8%) | 33406 (25.5%) | 10236 (30.2%) | 3703 (32.4%) | 154038 (31.7%) |
| Missing | 66542 (21.6%) | 43101 (24.2%) | 33931 (25.9%) | 6632 (19.5%) | 2071 (18.1%) | 109643 (22.6%) |
| **Body mass index,** **kg/m^2^** | 25.1 (3.0) | 31.5 (4.5) | 31.8 (4.6) | 30.6 (3.9) | 30.3 (4.0) | 27.4 (4.8) |
| **Waist circumference, cm** | 83.2 (9.4) | 102.6 (10.0) | 102.6 (10.2) | 102.1 (9.4) | 103.3 (9.9) | 90.3 (13.4) |
| **Waist-to-hip ratio, %** | 83.5 (7.6) | 93.6 (7.3) | 93.1 (7.5) | 94.5 (6.7) | 96.5 (6.2) | 87.2 (9.0) |
| **Waist-to-height ratio, %** | 49.7 (5.2) | 60.2 (6.1) | 60.6 (6.2) | 59.2 (5.5) | 59.3 (5.7) | 53.6 (7.5) |
| **Fat mass, kg** | 21.0 (6.8) | 31.3 (10.0) | 32.2 (10.4) | 29.3 (8.7) | 27.9 (8.4) | 24.8 (9.5) |
| **Fat-free mass, kg** | 49.3 (9.7) | 60.1 (11.1) | 59.2 (11.3) | 62.0 (10.5) | 64.4 (9.3) | 53.3 (11.5) |
| **Body fat percent, %** | 29.9 (8.1) | 34.1 (8.6) | 35.0 (8.8) | 32.0 (7.7) | 29.9 (6.3) | 31.4 (8.5) |
| **Systolic blood pressure, mmHg** | 135.2 (18.7) | 142.6 (17.6) | 141.6 (17.5) | 144.4 (17.3) | 147.9 (17.7) | 137.9 (18.6) |
| **Diastolic blood pressure, mmHg** | 80.3 (9.8) | 85.7 (9.8) | 85.1 (9.7) | 86.9 (9.6) | 88.7 (10.0) | 82.3 (10.1) |
| **Fasting time, hr** | 3.8 (2.4) | 3.9 (2.5) | 3.8 (2.4) | 3.9 (2.6) | 4.3 (3.3) | 3.8 (2.4) |
| **ALP, log_10_ U/L** | 1.8 (0.3) | 1.9 (0.1) | 1.9 (0.1) | 1.9 (0.1) | 1.9 (0.1) | 1.9 (0.2) |
| **ALT, log_10_ U/L** | 1.2 (0.3) | 1.4 (0.2) | 1.4 (0.2) | 1.5 (0.2) | 1.5 (0.2) | 1.3 (0.3) |
| **AST, log_10_ U/L** | 1.3 (0.2) | 1.4 (0.2) | 1.4 (0.1) | 1.4 (0.1) | 1.5 (0.2) | 1.4 (0.2) |
| **GGT, log_10_ U/L** | 1.3 (0.3) | 1.6 (0.3) | 1.6 (0.3) | 1.7 (0.3) | 1.9 (0.3) | 1.4 (0.3) |
| **Triglycerides, mmol/L** | 1.2 [0.7] | 2.1 [1.3] | 2.1 [1.3] | 2.1 [1.4] | 2.2 [1.5] | 1.4 [1.1] |
| **Cholesterol, mmol/L** | 5.3 (1.7) | 5.7 (1.2) | 5.7 (1.3) | 5.8 (1.2) | 5.9 (1.2) | 5.5 (1.5) |
| **HDL cholesterol, mmol/L** | 1.4 (0.6) | 1.2 (0.4) | 1.1 (0.4) | 1.2 (0.4) | 1.3 (0.5) | 1.3 (0.5) |
| **Direct LDL, mmol/L** | 3.3 (1.2) | 3.6 (0.9) | 3.6 (0.9) | 3.7 (0.9) | 3.6 (0.9) | 3.4 (1.1) |
| **Apolipoprotein A, g/L** | 1.4 (0.5) | 1.4 (0.4) | 1.3 (0.4) | 1.4 (0.4) | 1.5 (0.4) | 1.4 (0.5) |
| **Apolipoprotein B, g/L** | 1.0 (0.3) | 1.1 (0.3) | 1.1 (0.3) | 1.1 (0.3) | 1.1 (0.3) | 1.0 (0.3) |
| **Glucose, mmol/L** | 4.4 (1.7) | 5.0 (1.9) | 5.0 (2.0) | 4.9 (1.7) | 5.0 (1.8) | 4.6 (1.8) |
| **HbA1c, mmol/mol** | 34.0 (6.8) | 36.8 (9.8) | 37.2 (10.1) | 35.6 (8.4) | 35.5 (8.6) | 35.0 (8.1) |
| **CRP, mg/L** | 0.9 [1.4] | 2.1 [3.0] | 2.2 [3.1] | 1.9 [2.6] | 1.9 [2.5] | 1.2 [2.1] |
| **Vitamin D, nmol/L** | 46.6 [34.4] | 40.5 [29.0] | 40.0 [28.5] | 43.3 [30.0] | 39.4 [31.6] | 44.2 [32.8] |
| **Creatinine, umol/L** | 66.6 [19.0] | 74.9 [20.0] | 74.4 [20.6] | 76.4 [18.4] | 76.2 [16.9] | 69.6 [20.2] |
| **Total bilirubin, umol/L** | 7.8 [4.1] | 8.1 [4.1] | 7.9 [4.0] | 8.5 [4.1] | 8.8 [4.1] | 7.9 [4.1] |
| **Direct bilirubin, umol/L** | 1.4 [0.8] | 1.5 [0.8] | 1.4 [0.8] | 1.6 [0.8] | 1.7 [0.9] | 1.5 [0.8] |
| **Albumin, g/L** | 41.0 (10.5) | 42.8 (8.2) | 42.7 (8.1) | 43.2 (8.2) | 43.1 (8.3) | 41.7 (9.8) |
| **Urate, umol/L** | 268.2 (83.2) | 353.1 (78.2) | 345.3 (76.4) | 369.9 (76.8) | 392.3 (82.1) | 299.3 (91.1) |
| **Hemoglobin, g/dL** | 13.9 (1.2) | 14.6 (1.2) | 14.5 (1.3) | 14.8 (1.1) | 15.0 (1.1) | 14.2 (1.2) |
| **Red blood cell count, 10^12^ cells/L** | 4.4 (0.4) | 4.7 (0.4) | 4.7 (0.4) | 4.6 (0.4) | 4.6 (0.4) | 4.5 (0.4) |
| **Platelet, 10^9^ cells/L** | 253.7 (59.3) | 251.8 (61.1) | 254.4 (61.8) | 246.7 (58.0) | 240.2 (57.3) | 253.0 (60.0) |
| **White blood cell count, 10^9^ cells/L** | 6.6 (2.1) | 7.3 (2.0) | 7.3 (2.1) | 7.2 (1.9) | 7.2 (2.1) | 6.9 (2.1) |
| **Basophil cell count, 10^9^ cells/L** | 0.0 [0.0] | 0.0 [0.0] | 0.0 [0.0] | 0.0 [0.0] | 0.0 [0.0] | 0.0 [0.0] |
| **eosinophil cell count, 10^9^ cells/L** | 0.1 [0.1] | 0.2 [0.1] | 0.2 [0.1] | 0.2 [0.1] | 0.2 [0.1] | 0.1 [0.1] |
| **Lymphocyte count, 10^9^ cells/L** | 1.8 [0.7] | 2.0 [0.8] | 2.0 [0.8] | 1.9 [0.7] | 1.9 [0.8] | 1.9 [0.8] |
| **Monocyte count, 10^9^ cells/L** | 0.4 [0.2] | 0.5 [0.2] | 0.5 [0.2] | 0.5 [0.2] | 0.5 [0.2] | 0.4 [0.2] |

HbA1c: glycated haemoglobin. HDL: high density lipoprotein. LDL: low density lipoprotein. CRP: C-reactive protein. The cutoff values for low, intermediate and high FIB4 scores were 1.30 and 2.67 for people < 65 years old, and 2.00 and 2.67 for people >= 65 years old.

### Supplementary table 2: association between SLD, FIB4 score levels and all-cause and disease-specific mortality, stratified by sex

|  |  | SLD |  |  |  |  |
| --- | --- | --- | --- | --- | --- | --- |
|  | Non-SLD | All SLD | Low FIB4 | Intermediate FIB4 | High FIB4 | P for trend for FIB4 levels |
| **All-cause mortality** | **Female + Male** |  |  |  |  |  |
| Death, n | 21754 | 20766 | 12589 | 6271 | 1334 |  |
| Mortality rate, /1000 py | 5.25 | 8.78 | 7.89 | 9.57 | 25.01 |  |
| Rate difference, /1000py | Reference | 3.53 | 2.64 | 4.32 | 19.76 |  |
| Model 1: HR (95%CI) | Reference | 1.28 (1.26, 1.31) | 1.11 (1.09, 1.14) | 1.63 (1.58, 1.68) | 2.62 (2.48, 2.77) | <0.01 |
| Model 2: HR (95%CI) | Reference | 1.20 (1.18, 1.24) | 1.04 (1.01, 1.07) | 1.52 (1.47, 1.57) | 2.44 (2.30, 2.59) | <0.01 |
| Sensitivity analysis | Reference | 1.21 (1.18, 1.24) | 1.05 (1.02, 1.08) | 1.52 (1.47, 1.58) | 2.38 (2.25, 2.53) | <0.01 |
|  | **Male** |  |  |  |  |  |
| Death, n | 10545 | 14595 | 8290 | 4849 | 1077 |  |
| Mortality rate, /1000 py | 7.28 | 9.75 | 8.81 | 10.1 | 26.11 |  |
| Rate difference, /1000py | Reference | 2.47 | 1.53 | 2.82 | 18.83 |  |
| Model 1: HR (95%CI) | Reference | 1.18 (1.15, 1.21) | 1.00 (0.97, 1.03) | 1.45 (1.40, 1.50) | 2.32 (2.18, 2.47) | <0.01 |
| Model 2: HR (95%CI) | Reference | 1.24 (1.19, 1.28) | 1.04 (1.00, 1.08) | 1.50 (1.44, 1.56) | 2.35 (2.20, 2.51) | <0.01 |
| Sensitivity analysis | Reference | 1.23 (1.19, 1.27) | 1.04 (1.00, 1.08) | 1.50 (1.43, 1.56) | 2.30 (2.14, 2.46) | <0.01 |
|  | **Female** |  |  |  |  |  |
| Death, n | 11209 | 6171 | 4299 | 1422 | 257 |  |
| Mortality rate, /1000 py | 4.16 | 7.12 | 6.57 | 8.14 | 21.23 |  |
| Rate difference, /1000py | Reference | 2.96 | 2.41 | 3.98 | 17.07 |  |
| Model 1: HR (95%CI) | Reference | 1.45 (1.40, 1.50) | 1.28 (1.23, 1.32) | 2.04 (1.93, 2.16) | 3.51 (3.10, 3.97) | <0.01 |
| Model 2: HR (95%CI) | Reference | 1.21 (1.16, 1.25) | 1.07 (1.03, 1.11) | 1.67 (1.57, 1.77) | 2.91 (2.57, 3.30) | <0.01 |
| Sensitivity analysis | Reference | 1.22 (1.17, 1.27) | 1.08 (1.04, 1.13) | 1.69 (1.59, 1.80) | 2.81 (2.47, 3.21) | <0.01 |
| **Liver related mortality** | **Female + Male** |  |  |  |  |  |
| Death, n | 472 | 874 | 300 | 276 | 277 |  |
| Mortality rate, /1000 py | 0.11 | 0.37 | 0.19 | 0.42 | 5.19 |  |
| Rate difference, /1000py | Reference | 0.26 | 0.08 | 0.31 | 5.08 |  |
| Model 1: HR (95%CI) | Reference | 2.36 (2.09, 2.65) | 1.25 (1.07, 1.45) | 2.91 (2.49, 3.40) | 29.23 (24.86, 34.37) | <0.01 |
| Model 2: HR (95%CI) | Reference | 2.85 (2.45, 3.33) | 1.41 (1.18, 1.68) | 3.30 (2.74, 3.96) | 31.76 (26.44, 38.14) | <0.01 |
| Sensitivity analysis | Reference | 2.88 (2.46, 3.38) | 1.44 (1.20, 1.74) | 3.37 (2.79, 4.08) | 31.91 (26.36, 38.61) | <0.01 |
|  | Male |  |  |  |  |  |
| Death, n | 240 | 644 | 188 | 226 | 215 |  |
| Mortality rate, /1000 py | 0.17 | 0.43 | 0.2 | 0.47 | 5.21 |  |
| Rate difference, /1000py | Reference | 0.26 | 0.03 | 0.3 | 5.04 |  |
| Model 1: HR (95%CI) | Reference | 2.17 (1.87, 2.53) | 0.99 (0.81, 1.20) | 2.53 (2.10, 3.04) | 23.98 (19.80, 29.06) | <0.01 |
| Model 2: HR (95%CI) | Reference | 3.44 (2.80, 4.23) | 1.40 (1.11, 1.78) | 3.56 (2.83, 4.47) | 31.73 (25.31, 39.80) | <0.01 |
| Sensitivity analysis | Reference | 3.48 (2.81, 4.32) | 1.44 (1.12, 1.84) | 3.68 (2.90, 4.66) | 32.17 (25.38, 40.76) | <0.01 |
|  | Female |  |  |  |  |  |
| Death, n | 232 | 230 | 112 | 50 | 62 |  |
| Mortality rate, /1000 py | 0.09 | 0.27 | 0.17 | 0.29 | 5.12 |  |
| Rate difference, /1000py | Reference | 0.18 | 0.08 | 0.2 | 5.03 |  |
| Model 1: HR (95%CI) | Reference | 2.68 (2.23, 3.48) | 1.71 (1.36, 2.15) | 3.17 (2.33, 4.33) | 44.77 (33.46, 59.91) | <0.01 |
| Model 2: HR (95%CI) | Reference | 2.38 (1.89, 3.00) | 1.50 (1.15, 1.96) | 2.77 (1.97, 3.89) | 38.36 (27.89, 52.74) | <0.01 |
| Sensitivity analysis | Reference | 2.40 (1.89, 3.04) | 1.54 (1.18, 2.03) | 2.73 (1.91, 3.88) | 38.12 (27.39, 53.05) | <0.01 |
| **Extrahepatic cancer** | **Female + Male** |  |  |  |  |  |
| Death, n | 10573 | 8819 | 5588 | 2604 | 398 |  |
| Mortality rate, /1000 py | 2.55 | 3.73 | 3.5 | 3.98 | 7.46 |  |
| Rate difference, /1000py | Reference | 1.18 | 0.95 | 1.43 | 4.91 |  |
| Model 1: HR (95%CI) | Reference | 1.22 (1.19, 1.26) | 1.11 (1.07, 1.15) | 1.49 (1.42, 1.56) | 1.84 (1.66, 2.04) | <0.01 |
| Model 2: HR (95%CI) | Reference | 1.14 (1.10, 1.19) | 1.04 (1.00, 1.08) | 1.39 (1.32, 1.46) | 1.72 (1.55, 1.91) | <0.01 |
| Sensitivity analysis | Reference | 1.15 (1.10, 1.19) | 1.04 (1.00, 1.09) | 1.39 (1.32, 1.46) | 1.63 (1.46, 1.82) | <0.01 |
|  | Male |  |  |  |  |  |
| Death, n | 4465 | 5892 | 3522 | 1906 | 321 |  |
| Mortality rate, /1000 py | 3.08 | 3.93 | 3.74 | 3.97 | 7.78 |  |
| Rate difference, /1000py | Reference | 0.85 | 0.66 | 0.89 | 4.7 |  |
| Model 1: HR (95%CI) | Reference | 1.15 (1.10, 1.19) | 1.03 (0.99, 1.08) | 1.34 (1.27, 1.42) | 1.68 (1.50, 1.88) | <0.01 |
| Model 2: HR (95%CI) | Reference | 1.16 (1.10, 1.22) | 1.04 (0.98, 1.10) | 1.35 (1.26, 1.44) | 1.67 (1.48, 1.88) | <0.01 |
| Sensitivity analysis | Reference | 1.15 (1.09, 1.21) | 1.03 (0.97, 1.09) | 1.34 (1.25, 1.43) | 1.57 (1.39, 1.78) | <0.01 |
|  | Female |  |  |  |  |  |
| Death, n | 6108 | 2927 | 2066 | 698 | 77 |  |
| Mortality rate, /1000 py | 2.27 | 3.38 | 3.16 | 3.99 | 6.36 |  |
| Rate difference, /1000py | Reference | 1.11 | 0.89 | 1.72 | 4.09 |  |
| Model 1: HR (95%CI) | Reference | 1.33 (1.27, 1.39) | 1.20 (1.14, 1.26) | 1.83 (1.69, 1.98) | 2.10 (1.67, 2.63) | <0.01 |
| Model 2: HR (95%CI) | Reference | 1.15 (1.09, 1.21) | 1.04 (0.98, 1.10) | 1.56 (1.44, 1.70) | 1.81 (1.44, 2.27) | <0.01 |
| Sensitivity analysis | Reference | 1.16 (1.10, 1.23) | 1.06 (1.00, 1.13) | 1.57 (1.44, 1.71) | 1.70 (1.33, 2.16) | <0.01 |
| **CVD** | **Female + Male** |  |  |  |  |  |
| Death, n | 3860 | 4997 | 2977 | 1575 | 306 |  |
| Mortality rate, /1000 py | 0.93 | 2.11 | 1.87 | 2.4 | 5.74 |  |
| Rate difference, /1000py | Reference | 1.18 | 0.94 | 1.47 | 4.81 |  |
| Model 1: HR (95%CI) | Reference | 1.57 (1.50, 1.64) | 1.34 (1.28, 1.41) | 2.08 (1.96, 2.22) | 2.88 (2.56, 3.25) | <0.01 |
| Model 2: HR (95%CI) | Reference | 1.34 (1.27, 1.41) | 1.14 (1.07, 1.21) | 1.76 (1.64, 1.89) | 2.44 (2.16, 2.76) | <0.01 |
| Sensitivity analysis | Reference | 1.34 (1.27, 1.42) | 1.15 (1.08, 1.22) | 1.76 (1.64, 1.89) | 2.41 (2.12, 2.74) | <0.01 |
|  | Male |  |  |  |  |  |
| Death, n | 2281 | 3831 | 2151 | 1316 | 261 |  |
| Mortality rate, /1000 py | 1.57 | 2.56 | 2.29 | 2.74 | 6.33 |  |
| Rate difference, /1000py | Reference | 0.99 | 0.72 | 1.17 | 4.76 |  |
| Model 1: HR (95%CI) | Reference | 1.43 (1.36, 1.51) | 1.19 (1.12, 1.27) | 1.85 (1.73, 1.98) | 2.60 (2.28, 2.96) | <0.01 |
| Model 2: HR (95%CI) | Reference | 1.37 (1.28, 1.47) | 1.13 (1.05, 1.22) | 1.74 (1.61, 1.89) | 2.41 (2.11, 2.76) | <0.01 |
| Sensitivity analysis | Reference | 1.38 (1.28, 1.48) | 1.15 (1.06, 1.24) | 1.74 (1.60, 1.89) | 2.41 (2.10, 2.78) | <0.01 |
|  | Female |  |  |  |  |  |
| Death, n | 1579 | 1166 | 826 | 259 | 45 |  |
| Mortality rate, /1000 py | 0.59 | 1.35 | 1.26 | 1.48 | 3.72 |  |
| Rate difference, /1000py | Reference | 0.76 | 0.67 | 0.89 | 3.13 |  |
| Model 1: HR (95%CI) | Reference | 1.86 (1.72, 2.01) | 1.64 (1.50, 1.79) | 2.71 (2.37, 3.10) | 3.98 (2.96, 5.36) | <0.01 |
| Model 2: HR (95%CI) | Reference | 1.32 (1.20, 1.44) | 1.17 (1.06, 1.29) | 1.85 (1.61, 2.14) | 2.80 (2.07, 3.79) | <0.01 |
| Sensitivity analysis | Reference | 1.32 (1.20, 1.45) | 1.18 (1.07, 1.30) | 1.89 (1.64, 2.19) | 2.57 (1.86, 3.55) | <0.01 |

HR (95%CI): hazard ratio (95% confidence interval). SLD: steatotic liver disease. CVD: cardiovascular disease. HCC: hepatocellular carcinoma. Model 1 was stratified by region and age group (<65 vs. >=65) and adjusted for sex, ethnicity, education, Townsend Deprivation Index, physical activity level, smoking, drinking. Model 2 was additionally adjusted for cardiometabolic risk factors (hypertension, obesity, diabetes, high triglyceride, and low HDL-cholesterol). Sensitivity analysis excluded the first two years of follow-up.

### Supplementary table 3: sensitivity analyses for adjusted associations between SLD subtypes, FIB4 scores and all-cause and selected disease-specific mortality.

|  | Sensitivity analysis 1 | | |  | Sensitivity analysis 2 | | |
| --- | --- | --- | --- | --- | --- | --- | --- |
|  | Events | Mortality rate, /1000py | HR (95%CI) |  | Events | Mortality rate, /1000py | HR (95%CI) |
| **All-cause mortality** | | | |  |  |  |  |
| non-SLD | 21754 | 5.25 | Reference |  | 20535 | 4.96 | Reference |
| MASLD | 14910 | 8.58 | 1.20 (1.17, 1.24) |  | 14110 | 8.12 | 1.33 (1.30, 1.36) |
| FIB4 low | 9517 | 7.92 | 1.06 (1.03, 1.09) |  | 9035 | 7.51 | 1.16 (1.13, 1.19) |
| FIB4 intermediate | 4247 | 9.34 | 1.52 (1.47, 1.58) |  | 4005 | 8.81 | 1.65 (1.60, 1.71) |
| FIB4 high | 707 | 20.88 | 2.00 (1.85, 2.16) |  | 653 | 19.28 | 2.12 (1.96, 2.30) |
| MetALD | 4247 | 8.19 | 1.10 (1.06, 1.15) |  | 4005 | 7.72 | 1.17 (1.13, 1.21) |
| FIB4 low | 2113 | 7.21 | 0.93 (0.89, 0.98) |  | 1983 | 6.77 | 0.97 (0.93, 1.02) |
| FIB4 intermediate | 1246 | 9 | 1.39 (1.31, 1.48) |  | 1186 | 8.57 | 1.48 (1.39, 1.57) |
| FIB4 high | 268 | 25.9 | 2.44 (2.16, 2.76) |  | 251 | 24.26 | 2.56 (2.26, 2.90) |
| ALD-1 | 1664 | 11.1 | 1.25 (1.17, 1.34) |  | 1574 | 10.5 | 1.39 (1.32, 1.47) |
| FIB4 low | 759 | 8.87 | 1.00 (0.92, 1.09) |  | 725 | 8.47 | 1.09 (1.01, 1.17) |
| FIB4 intermediate | 630 | 11.6 | 1.56 (1.42, 1.70) |  | 599 | 11.02 | 1.73 (1.59, 1.88) |
| FIB4 high | 229 | 32.64 | 3.30 (2.87, 3.78) |  | 209 | 29.78 | 3.60 (3.14, 4.13) |
| **Mortality of extrahepatic cancer** | | | |  |  |  |  |
| non-SLD | 10573 | 2.55 | Reference |  | 9887 | 2.39 | Reference |
| MASLD | 1629 | 3.67 | 1.17 (1.12, 1.21) |  | 1526 | 3.43 | 1.26 (1.22, 1.31) |
| FIB4 low | 4155 | 3.46 | 1.05 (1.00, 1.09) |  | 3901 | 3.24 | 1.13 (1.09, 1.18) |
| FIB4 intermediate | 1827 | 4.02 | 1.45 (1.37, 1.54) |  | 1701 | 3.74 | 1.56 (1.48, 1.64) |
| FIB4 high | 213 | 6.29 | 1.45 (1.27, 1.67) |  | 186 | 5.49 | 1.46 (1.26, 1.69) |
| MetALD | 1629 | 3.6 | 1.07 (1.00, 1.14) |  | 1526 | 3.37 | 1.16 (1.10, 1.23) |
| FIB4 low | 1003 | 3.42 | 0.97 (0.91, 1.05) |  | 933 | 3.18 | 1.05 (0.98, 1.12) |
| FIB4 intermediate | 506 | 3.66 | 1.21 (1.10, 1.33) |  | 485 | 3.5 | 1.35 (1.23, 1.48) |
| FIB4 high | 88 | 8.5 | 1.85 (1.49, 2.28) |  | 77 | 7.44 | 1.88 (1.50, 2.36) |
| ALD-1 | 655 | 4.37 | 1.10 (0.99, 1.22) |  | 615 | 4.1 | 1.29 (1.19, 1.41) |
| FIB4 low | 345 | 4.03 | 1.00 (0.89, 1.13) |  | 329 | 3.84 | 1.17 (1.05, 1.31) |
| FIB4 intermediate | 226 | 4.16 | 1.20 (1.04, 1.39) |  | 213 | 3.92 | 1.41 (1.23, 1.62) |
| FIB4 high | 67 | 9.55 | 2.14 (1.66, 2.74) |  | 59 | 8.41 | 2.41 (1.86, 3.11) |
| **Mortality of cardiovascular diseases** | | | |  |  |  |  |
| non-SLD | 3860 | 0.93 | Reference |  | 3627 | 0.88 | Reference |
| MASLD | 906 | 2.08 | 1.34 (1.26, 1.42) |  | 844 | 1.94 | 1.65 (1.57, 1.73) |
| FIB4 low | 2272 | 1.89 | 1.17 (1.10, 1.25) |  | 2138 | 1.78 | 1.42 (1.35, 1.50) |
| FIB4 intermediate | 1047 | 2.3 | 1.72 (1.59, 1.86) |  | 970 | 2.13 | 2.06 (1.91, 2.22) |
| FIB4 high | 188 | 5.55 | 2.29 (1.97, 2.66) |  | 171 | 5.05 | 2.68 (2.29, 3.13) |
| MetALD | 906 | 2 | 1.27 (1.16, 1.39) |  | 844 | 1.87 | 1.42 (1.31, 1.53) |
| FIB4 low | 484 | 1.65 | 1.01 (0.90, 1.12) |  | 450 | 1.54 | 1.10 (0.99, 1.21) |
| FIB4 intermediate | 347 | 2.51 | 1.84 (1.63, 2.07) |  | 324 | 2.34 | 2.04 (1.81, 2.29) |
| FIB4 high | 58 | 5.61 | 2.38 (1.83, 3.10) |  | 55 | 5.32 | 2.67 (2.04, 3.49) |
| ALD | 400 | 2.67 | 1.40 (1.21, 1.61) |  | 374 | 2.49 | 1.54 (1.38, 1.73) |
| FIB4 low | 181 | 2.12 | 1.11 (0.93, 1.32) |  | 171 | 2 | 1.20 (1.03, 1.41) |
| FIB4 intermediate | 158 | 2.91 | 1.85 (1.54, 2.24) |  | 147 | 2.71 | 2.04 (1.73, 2.41) |
| FIB4 high | 45 | 6.41 | 2.99 (2.19, 4.08) |  | 41 | 5.84 | 3.30 (2.42, 4.49) |
| **Mortality of liver related diseases** | | | |  |  |  |  |
| non-SLD | 472 | 0.11 | Reference |  | 433 | 0.1 | Reference |
| MASLD | 164 | 0.26 | 2.09 (1.77, 2.47) |  | 157 | 0.24 | 2.01 (1.74, 2.31) |
| FIB4 low | 201 | 0.17 | 1.36 (1.12, 1.65) |  | 191 | 0.16 | 1.22 (1.03, 1.45) |
| FIB4 intermediate | 131 | 0.29 | 2.53 (2.03, 3.15) |  | 125 | 0.27 | 2.29 (1.87, 2.82) |
| FIB4 high | 109 | 3.22 | 22.17 (17.52, 28.05) |  | 103 | 3.04 | 20.86 (16.65, 26.13) |
| MetALD | 164 | 0.36 | 2.30 (1.86, 2.85) |  | 157 | 0.35 | 2.44 (2.01, 2.97) |
| FIB4 low | 54 | 0.18 | 1.25 (0.92, 1.69) |  | 52 | 0.18 | 1.26 (0.94, 1.69) |
| FIB4 intermediate | 55 | 0.4 | 2.85 (2.10, 3.85) |  | 52 | 0.38 | 2.87 (2.14, 3.86) |
| FIB4 high | 49 | 4.74 | 27.79 (20.26, 38.13) |  | 47 | 4.54 | 29.88 (21.89, 40.77) |
| ALD-1 | 166 | 1.11 | 3.74 (2.94, 4.75) |  | 156 | 1.04 | 5.32 (4.36, 6.50) |
| FIB4 low | 28 | 0.33 | 1.39 (0.91, 2.12) |  | 27 | 0.32 | 1.94 (1.30, 2.88) |
| FIB4 intermediate | 65 | 1.2 | 5.65 (4.19, 7.62) |  | 64 | 1.18 | 7.98 (6.07, 10.49) |
| FIB4 high | 69 | 9.83 | 39.56 (29.46, 53.10) |  | 61 | 8.69 | 56.24 (42.55, 74.35) |

HR (95%CI): hazard ratio (95% confidence interval). py: person-years. SLD: steatotic liver disease. CVD: cardiovascular disease. HCC: hepatocellular carcinoma. Model 1 stratified by region and age group (<65 vs. >=65) and adjusted for sex, ethnicity, education, Townsend Deprivation Index, physical activity level, smoking, and cardiometabolic risk factors (hypertension, obesity, diabetes, high triglyceride, and low HDL-cholesterol). The cutoff values for low, intermediate and high FIB4 scores were 1.30 and 2.67 for people < 65 years old, and 2.00 and 2.67 for people >= 65 years old. Sensitivity analysis 1: additional adjustment of alcohol drinking and cardiometabolic risk factors. Sensitivity analysis 2: removing the first two-years of follow-up.

### Supplementary table 4: Adjusted associations between HSI-defined SLD subtypes, FIB4 scores, and all-cause and cause-specific mortality.

|  | All-cuase mortality | |  | Mortality of extrahepatic cancer | |  | Mortality of CVD | |  | Mortality of liver-related diseases | |
| --- | --- | --- | --- | --- | --- | --- | --- | --- | --- | --- | --- |
|  | Events/total | HR (95%CI) |  | Events/total | HR (95%CI) |  | Events/total | HR (95%CI) |  | Events/total | HR (95%CI) |
| non-SLD | 21599 / 275124 | Reference |  | 10181 / 275124 | Reference |  | 3978 / 275124 | Reference |  | 588 / 275124 | Reference |
| MASLD | 15997 / 163467 | 1.23 (1.20, 1.26) |  | 7073 / 163467 | 1.17 (1.13, 1.21) |  | 3715 / 163467 | 1.58 (1.51, 1.65) |  | 443 / 163467 | 1.51 (1.32, 1.72) |
| FIB4 low | 10951 / 120380 | 1.04 (1.01, 1.06) |  | 4937 / 120380 | 1.02 (0.98, 1.05) |  | 2511 / 120380 | 1.31 (1.24, 1.37) |  | 221 / 120380 | 0.78 (0.67, 0.91) |
| FIB4 intermediate | 3979 / 35645 | 1.54 (1.49, 1.59) |  | 1744 / 35645 | 1.45 (1.38, 1.53) |  | 956 / 35645 | 2.01 (1.87, 2.16) |  | 131 / 35645 | 1.65 (1.36, 2.00) |
| FIB4 high | 506 / 2136 | 2.00 (1.83, 2.18) |  | 149 / 2136 | 1.38 (1.18, 1.63) |  | 122 / 2136 | 2.39 (1.99, 2.86) |  | 82 / 2136 | 14.19 (11.20, 17.97) |
| MetALD | 3337 / 35850 | 1.07 (1.03, 1.11) |  | 1534 / 35850 | 1.08 (1.02, 1.14) |  | 802 / 35850 | 1.33 (1.23, 1.44) |  | 135 / 35850 | 1.70 (1.40, 2.07) |
| FIB4 low | 2106 / 25564 | 0.87 (0.83, 0.91) |  | 1019 / 25564 | 0.93 (0.87, 0.99) |  | 487 / 25564 | 1.04 (0.95, 1.15) |  | 57 / 25564 | 0.82 (0.63, 1.08) |
| FIB4 intermediate | 980 / 8698 | 1.38 (1.30, 1.47) |  | 424 / 8698 | 1.31 (1.19, 1.44) |  | 251 / 8698 | 1.84 (1.61, 2.09) |  | 45 / 8698 | 1.96 (1.44, 2.67) |
| FIB4 high | 158 / 569 | 2.18 (1.86, 2.55) |  | 52 / 569 | 1.70 (1.30, 2.24) |  | 37 / 569 | 2.46 (1.78, 3.40) |  | 28 / 569 | 16.22 (11.05, 23.80) |
| ALD-1 | 1182 / 9359 | 1.22 (1.14, 1.29) |  | 469 / 9359 | 1.13 (1.03, 1.24) |  | 290 / 9359 | 1.42 (1.25, 1.60) |  | 105 / 9359 | 3.50 (2.82, 4.34) |
| FIB4 low | 640 / 5948 | 0.96 (0.89, 1.04) |  | 285 / 5948 | 1.00 (0.89, 1.13) |  | 153 / 5948 | 1.08 (0.92, 1.28) |  | 29 / 5948 | 1.40 (0.96, 2.05) |
| FIB4 intermediate | 398 / 2858 | 1.49 (1.35, 1.65) |  | 140 / 2858 | 1.20 (1.01, 1.42) |  | 108 / 2858 | 1.96 (1.62, 2.38) |  | 40 / 2858 | 4.42 (3.19, 6.11) |
| FIB4 high | 110 / 335 | 3.09 (2.56, 3.72) |  | 34 / 335 | 2.26 (1.61, 3.16) |  | 19 / 335 | 2.48 (1.58, 3.89) |  | 35 / 335 | 35.20 (24.92, 49.73) |

HSI: hepatic steatosis index. In this sensitivity analysis, presence of liver steatosis was defined as HSI > 36. HR (95%CI): hazard ratio (95% confidence interval). SLD: steatotic liver disease. CVD: cardiovascular disease. Model was stratified by region and age group (<65 vs. >=65) and adjusted for sex, ethnicity, education, Townsend Deprivation Index, physical activity level, smoking. The cutoff values for low, intermediate and high FIB4 scores were 1.30 and 2.67 for people < 65 years old, and 2.00 and 2.67 for people >= 65 years old.

### Supplementary table 5: Adjusted associations between SLD subtypes, FIB4 scores, and all-cause and cause-specific mortality using fatty liver index > 70 for liver steatosis.

|  | All-cuase mortality | |  | Mortality of extrahepatic cancer | |  | Mortality of CVD | |  | Mortality of liver-related diseases | |
| --- | --- | --- | --- | --- | --- | --- | --- | --- | --- | --- | --- |
|  | Events/total | HR (95%CI) |  | Events/total | HR (95%CI) |  | Events/total | HR (95%CI) |  | Events/total | HR (95%CI) |
| non-SLD | 25447 / 347379 | Reference |  | 12358 / 347379 | Reference |  | 4623 / 347379 | Reference |  | 554 / 347379 | Reference |
| MASLD | 12182 / 101058 | 1.37 (1.34, 1.40) |  | 5050 / 101058 | 1.26 (1.22, 1.30) |  | 3058 / 101058 | 1.76 (1.67, 1.84) |  | 401 / 101058 | 2.16 (1.89, 2.47) |
| FIB4 low | 7776 / 69763 | 1.19 (1.16, 1.22) |  | 3304 / 69763 | 1.13 (1.08, 1.17) |  | 1938 / 69763 | 1.51 (1.43, 1.60) |  | 171 / 69763 | 1.25 (1.05, 1.49) |
| FIB4 intermediate | 3448 / 26350 | 1.72 (1.66, 1.78) |  | 1435 / 26350 | 1.56 (1.47, 1.65) |  | 872 / 26350 | 2.19 (2.04, 2.36) |  | 124 / 26350 | 2.62 (2.15, 3.21) |
| FIB4 high | 587 / 2151 | 2.31 (2.12, 2.51) |  | 166 / 2151 | 1.54 (1.32, 1.80) |  | 160 / 2151 | 2.96 (2.53, 3.48) |  | 100 / 2151 | 22.90 (18.35, 28.58) |
| MetALD | 3031 / 26571 | 1.18 (1.14, 1.23) |  | 1299 / 26571 | 1.14 (1.08, 1.21) |  | 759 / 26571 | 1.45 (1.34, 1.57) |  | 147 / 26571 | 2.51 (2.07, 3.04) |
| FIB4 low | 1705 / 17110 | 0.98 (0.93, 1.03) |  | 793 / 17110 | 1.02 (0.95, 1.10) |  | 403 / 17110 | 1.12 (1.01, 1.24) |  | 45 / 17110 | 1.22 (0.89, 1.65) |
| FIB4 intermediate | 1016 / 8125 | 1.50 (1.41, 1.60) |  | 402 / 8125 | 1.30 (1.18, 1.44) |  | 290 / 8125 | 2.11 (1.87, 2.38) |  | 50 / 8125 | 3.10 (2.30, 4.16) |
| FIB4 high | 241 / 709 | 2.73 (2.40, 3.10) |  | 78 / 709 | 2.08 (1.66, 2.60) |  | 50 / 709 | 2.62 (1.98, 3.46) |  | 46 / 709 | 30.76 (22.57, 41.91) |
| ALD-1 | 1445 / 9597 | 1.39 (1.32, 1.47) |  | 551 / 9597 | 1.25 (1.14, 1.36) |  | 352 / 9597 | 1.56 (1.39, 1.74) |  | 153 / 9597 | 5.25 (4.33, 6.38) |
| FIB4 low | 644 / 5328 | 1.08 (1.00, 1.17) |  | 288 / 5328 | 1.12 (1.00, 1.26) |  | 155 / 5328 | 1.19 (1.02, 1.40) |  | 24 / 5328 | 1.81 (1.20, 2.74) |
| FIB4 intermediate | 547 / 3519 | 1.71 (1.57, 1.86) |  | 189 / 3519 | 1.33 (1.15, 1.54) |  | 141 / 3519 | 2.08 (1.75, 2.46) |  | 59 / 3519 | 7.60 (5.76, 10.03) |
| FIB4 high | 215 / 547 | 3.82 (3.33, 4.37) |  | 62 / 547 | 2.55 (1.99, 3.28) |  | 42 / 547 | 3.39 (2.50, 4.60) |  | 66 / 547 | 59.09 (45.34, 76.99) |

In this sensitivity analysis, presence of liver steatosis was defined as fatty liver index > 70. HR (95%CI): hazard ratio (95% confidence interval). SLD: steatotic liver disease. CVD: cardiovascular disease. Model was stratified by region and age group (<65 vs. >=65) and adjusted for sex, ethnicity, education, Townsend Deprivation Index, physical activity level, smoking. The cutoff values for low, intermediate and high FIB4 scores were 1.30 and 2.67 for people < 65 years old, and 2.00 and 2.67 for people >= 65 years old.

### Supplementary table 6: Adjusted associations between SLD subtypes, FIB4 scores, and all-cause and cause-specific mortality excluding participants with borderline alcohol consumption.

|  | All-cuase mortality | |  | Mortality of extrahepatic cancer | |  | Mortality of CVD | |  | Mortality of liver-related diseases | |
| --- | --- | --- | --- | --- | --- | --- | --- | --- | --- | --- | --- |
|  | Events/total | HR (95%CI) |  | Events/total | HR (95%CI) |  | Events/total | HR (95%CI) |  | Events/total | HR (95%CI) |
| non-SLD | 20566 / 288629 | Reference |  | 9940 / 288629 | Reference |  | 3659 / 288629 | Reference |  | 452 / 288629 | Reference |
| MASLD | 14350 / 125983 | 1.32 (1.29, 1.35) |  | 6125 / 125983 | 1.26 (1.22, 1.30) |  | 3480 / 125983 | 1.64 (1.56, 1.72) |  | 428 / 125983 | 1.89 (1.65, 2.18) |
| FIB4 low | 9173 / 86707 | 1.14 (1.12, 1.17) |  | 4001 / 86707 | 1.12 (1.08, 1.16) |  | 2194 / 86707 | 1.41 (1.33, 1.49) |  | 193 / 86707 | 1.15 (0.97, 1.37) |
| FIB4 intermediate | 4083 / 33213 | 1.64 (1.58, 1.70) |  | 1750 / 33213 | 1.55 (1.47, 1.63) |  | 1006 / 33213 | 2.06 (1.92, 2.22) |  | 126 / 33213 | 2.18 (1.77, 2.67) |
| FIB4 high | 679 / 2640 | 2.19 (2.02, 2.36) |  | 210 / 2640 | 1.61 (1.40, 1.85) |  | 181 / 2640 | 2.78 (2.39, 3.23) |  | 103 / 2640 | 19.89 (15.89, 24.91) |
| MetALD | 3123 / 28392 | 1.16 (1.11, 1.20) |  | 1371 / 28392 | 1.15 (1.09, 1.22) |  | 770 / 28392 | 1.41 (1.30, 1.52) |  | 137 / 28392 | 2.27 (1.85, 2.78) |
| FIB4 low | 1774 / 18149 | 0.96 (0.92, 1.01) |  | 838 / 18149 | 1.04 (0.97, 1.12) |  | 412 / 18149 | 1.10 (0.99, 1.22) |  | 45 / 18149 | 1.15 (0.85, 1.58) |
| FIB4 intermediate | 1062 / 8837 | 1.45 (1.36, 1.54) |  | 431 / 8837 | 1.31 (1.19, 1.44) |  | 297 / 8837 | 2.02 (1.79, 2.28) |  | 49 / 8837 | 2.83 (2.09, 3.83) |
| FIB4 high | 226 / 731 | 2.53 (2.22, 2.89) |  | 76 / 731 | 2.02 (1.61, 2.53) |  | 48 / 731 | 2.51 (1.89, 3.35) |  | 40 / 731 | 26.62 (19.08, 37.14) |
| ALD-1 | 1569 / 10540 | 1.37 (1.30, 1.44) |  | 606 / 10540 | 1.26 (1.16, 1.37) |  | 383 / 10540 | 1.54 (1.38, 1.72) |  | 157 / 10540 | 5.01 (4.10, 6.12) |
| FIB4 low | 701 / 5862 | 1.06 (0.98, 1.14) |  | 317 / 5862 | 1.13 (1.01, 1.27) |  | 169 / 5862 | 1.17 (1.00, 1.37) |  | 24 / 5862 | 1.64 (1.08, 2.49) |
| FIB4 intermediate | 604 / 3879 | 1.70 (1.57, 1.85) |  | 210 / 3879 | 1.36 (1.19, 1.57) |  | 155 / 3879 | 2.07 (1.76, 2.44) |  | 63 / 3879 | 7.36 (5.59, 9.69) |
| FIB4 high | 219 / 570 | 3.68 (3.22, 4.21) |  | 62 / 570 | 2.46 (1.92, 3.16) |  | 43 / 570 | 3.29 (2.43, 4.44) |  | 67 / 570 | 57.08 (43.61, 74.72) |

Borderline alcohol consumption level was defined as alcohol intake level lying +/- 2 g/day around the cutoff values (30 and 60 for males and 20 and 50 for females), which is 28-32 g/day and 58-62 g/day for males and 18-22 g/day and 48-52 g/day for females. HR (95%CI): hazard ratio (95% confidence interval). SLD: steatotic liver disease. CVD: cardiovascular disease. Model was stratified by region and age group (<65 vs. >=65) and adjusted for sex, ethnicity, education, Townsend Deprivation Index, physical activity level, smoking. The cutoff values for low, intermediate and high FIB4 scores were 1.30 and 2.67 for people < 65 years old, and 2.00 and 2.67 for people >= 65 years old.

### Supplementary table 7: Adjusted associations between SLD subtypes, FIB4 scores, and all-cause and cause-specific mortality with additional adjustment of alcohol intake.

|  | All-cuase mortality | |  | Mortality of extrahepatic cancer | |  | Mortality of CVD | |  | Mortality of liver-related diseases | |
| --- | --- | --- | --- | --- | --- | --- | --- | --- | --- | --- | --- |
|  | Events/total | HR (95%CI) |  | Events/total | HR (95%CI) |  | Events/total | HR (95%CI) |  | Events/total | HR (95%CI) |
| non-SLD | 21754 / 307820 | Reference |  | 10573 / 307820 | Reference |  | 3860 / 307820 | Reference |  | 472 / 307820 | Reference |
| MASLD | 14910 / 131020 | 1.32 (1.29, 1.35) |  | 6372 / 131020 | 1.27 (1.23, 1.31) |  | 3612 / 131020 | 1.62 (1.54, 1.70) |  | 447 / 131020 | 2.05 (1.79, 2.36) |
| FIB4 low | 9517 / 90035 | 1.15 (1.12, 1.18) |  | 4155 / 90035 | 1.13 (1.09, 1.17) |  | 2272 / 90035 | 1.39 (1.32, 1.47) |  | 201 / 90035 | 1.25 (1.06, 1.48) |
| FIB4 intermediate | 4247 / 34669 | 1.64 (1.59, 1.70) |  | 1827 / 34669 | 1.57 (1.49, 1.65) |  | 1047 / 34669 | 2.05 (1.90, 2.20) |  | 131 / 34669 | 2.33 (1.91, 2.84) |
| FIB4 high | 707 / 2766 | 2.16 (2.00, 2.33) |  | 213 / 2766 | 1.56 (1.36, 1.79) |  | 188 / 2766 | 2.71 (2.34, 3.14) |  | 109 / 2766 | 21.37 (17.16, 26.60) |
| MetALD | 3704 / 33945 | 1.16 (1.11, 1.20) |  | 1629 / 33945 | 1.13 (1.07, 1.20) |  | 906 / 33945 | 1.46 (1.35, 1.58) |  | 164 / 33945 | 2.08 (1.72, 2.52) |
| FIB4 low | 2113 / 21825 | 0.97 (0.92, 1.02) |  | 1003 / 21825 | 1.02 (0.95, 1.09) |  | 484 / 21825 | 1.13 (1.02, 1.25) |  | 54 / 21825 | 1.06 (0.80, 1.42) |
| FIB4 intermediate | 1246 / 10465 | 1.45 (1.37, 1.54) |  | 506 / 10465 | 1.28 (1.16, 1.40) |  | 347 / 10465 | 2.09 (1.86, 2.34) |  | 55 / 10465 | 2.45 (1.84, 3.26) |
| FIB4 high | 268 / 861 | 2.55 (2.26, 2.88) |  | 88 / 861 | 1.94 (1.57, 2.39) |  | 58 / 861 | 2.69 (2.07, 3.50) |  | 49 / 861 | 24.95 (18.40, 33.83) |
| ALD-1 | 1664 / 11437 | 1.36 (1.27, 1.45) |  | 655 / 11437 | 1.19 (1.07, 1.31) |  | 400 / 11437 | 1.69 (1.47, 1.94) |  | 166 / 11437 | 3.38 (2.72, 4.21) |
| FIB4 low | 759 / 6425 | 1.06 (0.97, 1.15) |  | 345 / 6425 | 1.06 (0.94, 1.20) |  | 181 / 6425 | 1.29 (1.08, 1.53) |  | 28 / 6425 | 1.20 (0.80, 1.80) |
| FIB4 intermediate | 630 / 4162 | 1.68 (1.53, 1.83) |  | 226 / 4162 | 1.29 (1.11, 1.49) |  | 158 / 4162 | 2.22 (1.85, 2.68) |  | 65 / 4162 | 4.85 (3.64, 6.45) |
| FIB4 high | 229 / 610 | 3.61 (3.15, 4.14) |  | 67 / 610 | 2.31 (1.80, 2.97) |  | 45 / 610 | 3.65 (2.67, 4.98) |  | 69 / 610 | 35.74 (26.91, 47.49) |

HR (95%CI): hazard ratio (95% confidence interval). SLD: steatotic liver disease. CVD: cardiovascular disease. Model was stratified by region and age group (<65 vs. >=65) and adjusted for sex, ethnicity, education, Townsend Deprivation Index, physical activity level, smoking and daily alcohol consumption. The cutoff values for low, intermediate and high FIB4 scores were 1.30 and 2.67 for people < 65 years old, and 2.00 and 2.67 for people >= 65 years old.

### Supplementary table 8: Adjusted associations between SLD subtypes, and cause-specific mortality estimated with Fine-Gray model.

|  | Mortality of extrahepatic cancer | |  | Mortality of CVD | |
| --- | --- | --- | --- | --- | --- |
|  | Events/total | HR (95%CI) |  | Events/total | HR (95%CI) |
| non-SLD | 10181 / 275124 | Reference |  | 3978 / 275124 | Reference |
| MASLD | 7073 / 163467 | 1.25 (1.21, 1.29) |  | 3715 / 163467 | 1.64 (1.57, 1.72) |
| MetALD | 1534 / 35850 | 1.15 (1.08, 1.22) |  | 802 / 35850 | 1.41 (1.31, 1.51) |
| ALD | 469 / 9359 | 1.24 (1.15, 1.34) |  | 290 / 9359 | 1.48 (1.33, 1.66) |

In this sensitivity analysis, associations were estimated with Fine-Gray model. HR (95%CI): hazard ratio (95% confidence interval). SLD: steatotic liver disease. CVD: cardiovascular disease. Model was stratified by region and age group (<65 vs. >=65) and adjusted for sex, ethnicity, education, Townsend Deprivation Index, physical activity level, smoking. The cutoff values for low, intermediate and high FIB4 scores were 1.30 and 2.67 for people < 65 years old, and 2.00 and 2.67 for people >= 65 years old.

### Supplementary table 9: Adjusted associations between SLD subtypes, FIB4 scores and all-cause and selected disease-specific mortality in males and females.

|  | Males | | |  | Females | | |  | RHR  (males: females) |
| --- | --- | --- | --- | --- | --- | --- | --- | --- | --- |
|  | Events | Mortality rate, /1000pys | HR (95%CI) |  | Events | Mortality rate, /1000pys | HR (95%CI) |  |  |
| All-cause mortality | |  |  |  |  |  |  |  |  |
| non-SLD | 10545 | 7.28 | Reference |  | 11209 | 4.16 | Reference |  |  |
| MASLD | 9649 | 9.6 | 1.20 (1.17, 1.23) |  | 5261 | 7.19 | 1.51 (1.46, 1.56) |  | 0.82 (0.79, 0.86) |
| FIB4 low | 5778 | 8.94 | 1.04 (1.00, 1.07) |  | 3739 | 6.73 | 1.30 (1.26, 1.35) |  | 0.82 (0.78, 0.86) |
| FIB4 intermediate | 3067 | 9.9 | 1.46 (1.40, 1.52) |  | 1180 | 8.14 | 2.05 (1.93, 2.18) |  | 0.73 (0.68, 0.78) |
| FIB4 high | 534 | 21.7 | 1.88 (1.72, 2.05) |  | 173 | 18.68 | 3.01 (2.59, 3.50) |  | 0.65 (0.55, 0.77) |
| MetALD | 3067 | 8.96 | 1.09 (1.04, 1.13) |  | 1180 | 5.81 | 1.27 (1.17, 1.38) |  | 0.89 (0.81, 0.98) |
| FIB4 low | 1691 | 8.04 | 0.90 (0.86, 0.95) |  | 422 | 5.1 | 1.05 (0.95, 1.16) |  | 0.89 (0.80, 0.99) |
| FIB4 intermediate | 1072 | 9.34 | 1.33 (1.25, 1.41) |  | 174 | 7.36 | 1.84 (1.58, 2.14) |  | 0.75 (0.64, 0.88) |
| FIB4 high | 235 | 27.5 | 2.32 (2.04, 2.65) |  | 33 | 18.32 | 3.16 (2.25, 4.45) |  | 0.78 (0.54, 1.12) |
| ALD | 1535 | 11.38 | 1.26 (1.19, 1.33) |  | 129 | 8.59 | 1.80 (1.50, 2.15) |  | 0.73 (0.60, 0.88) |
| FIB4 low | 692 | 9.17 | 0.97 (0.90, 1.05) |  | 67 | 6.65 | 1.41 (1.11, 1.79) |  | 0.71 (0.55, 0.92) |
| FIB4 intermediate | 594 | 11.82 | 1.56 (1.44, 1.70) |  | 36 | 8.82 | 2.07 (1.49, 2.87) |  | 0.78 (0.56, 1.09) |
| FIB4 high | 208 | 31.94 | 3.21 (2.80, 3.68) |  | 21 | 41.75 | 8.37 (5.45, 12.85) |  | 0.41 (0.26, 0.64) |
| Mortality of extrahepatic cancer | | |  |  |  |  |  |  |  |
| non-SLD | 4465 | 3.08 | Reference |  | 6108 | 2.27 | Reference |  |  |
| MASLD | 1287 | 3.88 | 1.17 (1.12, 1.23) |  | 342 | 3.38 | 1.37 (1.31, 1.44) |  | 0.89 (0.84, 0.95) |
| FIB4 low | 2402 | 3.72 | 1.05 (1.00, 1.10) |  | 1753 | 3.15 | 1.20 (1.14, 1.27) |  | 0.91 (0.85, 0.98) |
| FIB4 intermediate | 1238 | 4 | 1.40 (1.31, 1.49) |  | 589 | 4.06 | 1.88 (1.72, 2.05) |  | 0.75 (0.67, 0.83) |
| FIB4 high | 162 | 6.58 | 1.41 (1.20, 1.65) |  | 51 | 5.51 | 1.80 (1.36, 2.37) |  | 0.85 (0.62, 1.17) |
| MetALD | 1287 | 3.77 | 1.09 (1.02, 1.16) |  | 342 | 3.08 | 1.25 (1.12, 1.40) |  | 0.91 (0.80, 1.03) |
| FIB4 low | 764 | 3.63 | 0.98 (0.90, 1.05) |  | 239 | 2.89 | 1.11 (0.98, 1.27) |  | 0.92 (0.79, 1.07) |
| FIB4 intermediate | 420 | 3.66 | 1.21 (1.09, 1.34) |  | 86 | 3.64 | 1.62 (1.31, 2.01) |  | 0.76 (0.60, 0.96) |
| FIB4 high | 78 | 9.13 | 1.87 (1.49, 2.34) |  | 10 | 5.55 | 1.83 (0.98, 3.40) |  | 1.12 (0.58, 2.18) |
| ALD | 600 | 4.45 | 1.19 (1.09, 1.30) |  | 55 | 3.66 | 1.47 (1.12, 1.91) |  | 0.84 (0.63, 1.11) |
| FIB4 low | 308 | 4.08 | 1.04 (0.93, 1.17) |  | 37 | 3.67 | 1.43 (1.03, 1.98) |  | 0.76 (0.54, 1.07) |
| FIB4 intermediate | 216 | 4.3 | 1.34 (1.17, 1.54) |  | 10 | 2.45 | 1.02 (0.55, 1.90) |  | 1.34 (0.71, 2.53) |
| FIB4 high | 59 | 9.06 | 2.17 (1.67, 2.80) |  | 8 | 15.9 | 5.99 (2.99, 11.99) |  | 0.39 (0.19, 0.82) |
| Mortality of CVD | | |  |  |  |  |  |  |  |
| non-SLD | 2281 | 1.57 | Reference |  | 1579 | 0.59 | Reference |  |  |
| MASLD | 800 | 2.57 | 1.48 (1.40, 1.57) |  | 106 | 1.4 | 2.00 (1.84, 2.17) |  | 0.75 (0.68, 0.83) |
| FIB4 low | 1531 | 2.37 | 1.25 (1.17, 1.34) |  | 741 | 1.33 | 1.73 (1.58, 1.89) |  | 0.73 (0.65, 0.81) |
| FIB4 intermediate | 829 | 2.68 | 1.84 (1.70, 1.99) |  | 218 | 1.5 | 2.76 (2.39, 3.18) |  | 0.71 (0.60, 0.84) |
| FIB4 high | 154 | 6.26 | 2.49 (2.11, 2.93) |  | 34 | 3.67 | 3.75 (2.67, 5.28) |  | 0.63 (0.43, 0.92) |
| MetALD | 800 | 2.34 | 1.32 (1.22, 1.44) |  | 106 | 0.95 | 1.50 (1.23, 1.83) |  | 0.94 (0.75, 1.16) |
| FIB4 low | 420 | 2 | 1.03 (0.93, 1.15) |  | 64 | 0.77 | 1.13 (0.88, 1.46) |  | 0.95 (0.73, 1.25) |
| FIB4 intermediate | 314 | 2.74 | 1.82 (1.62, 2.05) |  | 33 | 1.4 | 2.66 (1.88, 3.75) |  | 0.76 (0.53, 1.10) |
| FIB4 high | 52 | 6.08 | 2.36 (1.79, 3.11) |  | 6 | 3.33 | 4.08 (1.83, 9.10) |  | 0.60 (0.26, 1.41) |
| ALD | 380 | 2.82 | 1.41 (1.26, 1.57) |  | 20 | 1.33 | 1.79 (1.11, 2.90) |  | 0.84 (0.52, 1.38) |
| FIB4 low | 171 | 2.27 | 1.08 (0.92, 1.26) |  | 10 | 0.99 | 1.52 (0.82, 2.83) |  | 0.76 (0.40, 1.44) |
| FIB4 intermediate | 153 | 3.04 | 1.84 (1.56, 2.17) |  | 5 | 1.22 | 2.17 (0.90, 5.23) |  | 0.93 (0.38, 2.27) |
| FIB4 high | 43 | 6.6 | 3.00 (2.22, 4.06) |  | 2 | 3.98 | 5.70 (1.42, 22.84) |  | 0.58 (0.14, 2.39) |
| Mortality of liver related diseases | | |  |  |  |  |  |  |  |
| non-SLD | 240 | 0.17 | Reference |  | 232 | 0.09 | Reference |  | 0.69 (0.53, 0.91) |
| MASLD | 137 | 0.28 | 1.63 (1.37, 1.95) |  | 27 | 0.23 | 2.40 (1.95, 2.96) |  | 0.59 (0.42, 0.82) |
| FIB4 low | 112 | 0.17 | 0.90 (0.72, 1.13) |  | 89 | 0.16 | 1.58 (1.23, 2.03) |  | 0.71 (0.46, 1.08) |
| FIB4 intermediate | 96 | 0.31 | 1.81 (1.42, 2.29) |  | 35 | 0.24 | 2.72 (1.89, 3.89) |  | 0.38 (0.25, 0.59) |
| FIB4 high | 71 | 2.89 | 14.67 (11.18, 19.26) |  | 38 | 4.1 | 36.65 (25.75, 52.17) |  | 0.88 (0.56, 1.39) |
| MetALD | 137 | 0.4 | 2.08 (1.67, 2.59) |  | 27 | 0.24 | 2.48 (1.66, 3.71) |  | 0.54 (0.29, 1.00) |
| FIB4 low | 39 | 0.19 | 0.92 (0.65, 1.29) |  | 15 | 0.18 | 1.81 (1.07, 3.05) |  | 0.91 (0.38, 2.17) |
| FIB4 intermediate | 49 | 0.43 | 2.33 (1.71, 3.18) |  | 6 | 0.25 | 2.79 (1.24, 6.30) |  | 0.80 (0.33, 1.91) |
| FIB4 high | 43 | 5.03 | 24.40 (17.52, 33.98) |  | 6 | 3.33 | 30.05 (13.30, 67.92) |  | 0.52 (0.30, 0.89) |
| ALD | 148 | 1.1 | 4.24 (3.42, 5.25) |  | 18 | 1.2 | 9.33 (5.65, 15.42) |  | 0.41 (0.14, 1.21) |
| FIB4 low | 24 | 0.32 | 1.40 (0.92, 2.13) |  | 4 | 0.4 | 3.77 (1.40, 10.16) |  | 0.43 (0.18, 1.01) |
| FIB4 intermediate | 59 | 1.17 | 5.74 (4.30, 7.66) |  | 6 | 1.47 | 14.70 (6.51, 33.23) |  | 0.35 (0.15, 0.77) |
| FIB4 high | 62 | 9.52 | 44.48 (33.51, 59.05) |  | 7 | 13.92 | 147.80 (69.01, 316.57) |  | 0.69 (0.53, 0.91) |

HR (95%CI): hazard ratio (95% confidence interval). RHR: ratio of HR, with males as reference, estimated by fitting an interaction term with sex in the adjusted Cox model. py: person-years. SLD: steatotic liver disease. CVD: cardiovascular disease. Model was stratified by region and age group (<65 vs. >=65) and adjusted for ethnicity, education, Townsend Deprivation Index, physical activity level, smoking. The cutoff values for low, intermediate and high FIB4 scores were 1.30 and 2.67 for people < 65 years old, and 2.00 and 2.67 for people >= 65 years old.
